# Fruit nutritional composition, antioxidant and biochemical profiling of diverse tomato *(Solanum lycopersicum L.)* genetic resource

**DOI:** 10.3389/fpls.2022.1035163

**Published:** 2022-10-13

**Authors:** Bushra Raza, Amjad Hameed, Muhammad Yussouf Saleem

**Affiliations:** Nuclear Institute for Agriculture and Biology College, Pakistan Institute of Engineering and Applied Sciences (NIAB-C, PIEAS), Faisalabad, Pakistan

**Keywords:** tomato, antioxidants, bioactive compounds, nutritional quality, determinate, indeterminate

## Abstract

Tomato is the second most important vegetable crop consumed globally, by the virtue of its antioxidant-rich phytochemicals and bioactive compounds. Identifying genotypes with high antioxidant capacities and nutritionally rich phytochemicals is imperative for improving human health. The present study aimed to analyze 21 antioxidant and nutritional compounds in 93 geographically diverse, high yielding, better quality, stress tolerant tomato genotypes (hybrids, parental lines, inbred lines, and advanced lines). Significant variation (p < 0.05) was detected for investigated traits among the tested genotypes. Principal component analysis revealed the hybrids NIAB-Jauhar, Iron-lady F1, NBH-258, Ahmar F1, NIAB-Gohar, the parents H-24, B-25, AVTO1080, Astra and AVTO1003, as well as the lines LBR-17, AVTO1315, AVTO1311 and Lyp-1 revealed superior performance for the traits such as chlorophylls, lycopene, total carotenoids, total antioxidant capacity, total oxidant status, protease, alpha-amylase and total flavonoid content. Whereas the hybrids Surkhail F1, NBH-204, NBH-229, NBH-151, NBH-196, NBH-152, NBH-261, NBH-228, NIAB-Jauhar, NBH-256 and NBH-255, the lines 21354, AVTO1315, Newcherry, LA4097, AVTO1311 and UAF-1 together with the parents Naqeeb, NCEBR-5, M-82 and LBR-10 exhibited significant contribution to the traits such as total soluble sugars, reducing sugars, malondialdehyde, ascorbic acid, esterase, peroxidase and superoxide dismutase. Moreover, the semi-determinate and determinate tomato genotypes together with the categories parent and line with positive factor scores of 3.184, 0.015, 0.325 and 0.186 in PC- I, exhibited better performance for the trait such as total chlorophylls, lycopene, total carotenoids, total oxidant status, protease, alpha-amylase, total antioxidant capacity, esterase and total flavonoid content. Whereas again the semi-determinate and indeterminate tomato genotypes along with the category hybrid with positive factor scores of 2.619, 0.252 and 0.114 in PC- II, exhibited better performance for the traits such as total soluble sugars, reducing sugars, chlorophyll b, malondialdehyde content, ascorbic acid, superoxide dismutase and peroxidase. Hybrid vigor was observed in the hybrids for investigated traits. The aforementioned tomato genotypes showing outstanding performance in the respective traits can be exploited in the breeding programs to improve nutritional quality of tomato that can further improve human health.

## Introduction

Horticultural plants including tomato *(Solanum lycopersicum L.)* have gained more popularity in recent years. They contain high amount of bioactive compounds such as flavonoids, phenolics, anthocyanins, phenolic acids as well as important nutritive compounds such as sugars, essential oils, carotenoids, vitamins, and minerals. Horticulture plants have a distinct flavor, taste, together with excellent medicinal value and health care functions (Dogan et al., 2014; Grygorieva et al., 2021; Saran et al., 2021). Tomato, which is an essential part of the Mediterranean diet, plays a pivotal role in human nutrition. It has been known as a potential source of bioactive compounds, exhibiting antimicrobial, anti-mutagenic, anti-inflammatory and anti-carcinogenic properties (García-Hernández et al., 2018; Navarro-González et al., 2018; Uçan and Uğur, 2021). Its effects are correlated to antioxidant activity of carotenoids (lycopene and β carotene) and various phenolic compounds (flavonoids and phenolic acids) (Coyago-Cruz et al., 2019). In human nutrition tomato plays a significant role because of its health-promoting benefits (Salehi et al., 2019). Tomato fruit is a reservoir of minerals, proteins, vitamins, essential amino acids, monounsaturated fatty acids and phytosterols (Elbadrawy and Sello, 2016). Lycopene is considered a major compound contributing 80-90% of the total carotenoid content (Nguyen and Schwartz, 1999), while β-carotene contributes about 7-10% to the total carotenoids content in tomato fruit (Frusciante et al., 2007). Lycopene exhibits a maximum singlet oxygen quenching rate and has strong antioxidant properties (Di Mascio et al., 1989). However, β-carotene is associated with provitamin A activity (Sies, 1991). Tomato fruit is a healthy source of bioactive molecules, including ascorbic acid and tocopherol (Beecher, 1998; Raffo et al., 2002). More than two billion people around the world are presently reported to be influenced by “hidden hunger” (lack of minerals and vitamins). Tomatoes along with oranges are a major source of vitamin C in many countries of the world (Proteggente et al., 2002).

The human body needs an appropriate balance between antioxidants and free radicals to maintain homeostasis. Free radicals are naturally produced in the body by various exogenous and endogenous sources, resulting in oxidative damage to the molecules (Khalid and Hameed, 2017). There are several endogenous sources of oxidants including mitochondrial respiratory chain, immune reaction and enzymes such as nitric oxide synthase and xanthine oxidase. Inadequate amount of nutrients intake in daily diet may also result in oxidative stress, damaging cellular defense mechanism. Macromolecules particularly protein, lipids and DNA are the natural target of oxidative stress. Antioxidant properties of tomato fruit are attributed to enzymes that can inhibit the multiplication of free radicals, resulting in a positive impact on a human diet (Borguini and Ferraz Da Silva Torres, 2009; Ulewicz-Magulska and Wesolowski, 2019). In plants enzymatic and non-enzymatic antioxidants overcome oxidative stresses. Enzymatic antioxidants particularly superoxide dismutase, catalase, ascorbate peroxidase and peroxidase have the ability to eliminate hydrogen peroxide and free radicals in the mitochondria as well as the chloroplast (Lee et al., 2007). Non-enzymatic antioxidants include two classes i.e., antioxidant related with the membrane that is lipid-soluble like beta carotene and alpha-tocopherol and the second class include water-soluble reducer such as phenolics, ascorbate and glutathione (Jaleel et al., 2009).

The antioxidant molecules involve in the living organism defense system works at different level. These levels may include prevention, radical scavenging and radical induced damage repair. Based on the line of defense, these antioxidants are grouped into three different levels. The enzymes superoxide dismutase, catalase, glutathione peroxidase, ascorbate peroxidase and peroxidase are considered the first line of defense against reactive oxygen species. These enzymes dismutate superoxide radical, breakdown hydroperoxides and hydrogen peroxides H_2_O_2_ to harmless molecules (alcohol/water and O_2_). They have a preventive role (prevents free radical formation). The second line defense antioxidants such as ascorbic acid and alpha tocopherol are involve in scavenging active radical to control chain propagation reaction by producing lesser damaging molecules in human body. The antioxidants included in the third line defense group works when free radical damage has already occurred. These enzymes repair DNA, lipids and proteins. They identify the damaged oxidized DNA, protein and lipids and prevent their accumulation to protect toxic effects in the human body. This group includes proteolytic enzymes (proteases) and DNA repair enzyme systems (glycosylases polymerases and nucleases) (Ighodaro and Akinloye, 2018). Whereas, malondialdehyde level in human body is commonly used as a marker of oxidative stress (Gaweł et al., 2004). Moreover, Superoxide dismutase (SODs) serves as an excellent therapeutic and anti-inflammatory agent against diseases caused by reactive oxygen species (Noor et al., 2002; Yasui and Baba, 2006; Younus, 2018).

Nutritional quality and flavor have been adversely affected during the period of domestication and progress of the cultivated tomato, *Solanum lycopersicum* (Aono et al., 2021). The nutritional and physiochemical properties of tomato differ on the bases of its cultivar and prevailing environmental conditions (Anza et al., 2006; Ali et al., 2021). Moreover, many crop species have been modified genetically to enhance productivity, quality and resistance to intrinsic and extrinsic damages (Asensio et al., 2019). As a result, crop varieties differ in their secondary metabolites profile, which is responsible for biological defense mechanism and stage differentiation. They are important aspects to be taken into account in the determination of the role of crops in human nutrition and health (Huang et al., 2005). Moreover, different varieties of tomato are not considered in the present nutritional databases. Although it is most likely that different varieties of tomato might exhibit important differences in their nutritional qualities and bioactive compounds (Anza et al., 2006). Significant efforts are required to explore the nutritional potential of important crops (Vats et al., 2020). Recent years has proved to enhance awareness of the significance of antioxidant in daily intake. As a result, the development of crop varieties with better nutritional value and antioxidant properties has now become the main concern.

The present study aimed to identify nutritional, antioxidant and biochemical composition of diverse tomato germplasm including hybrids, parental lines, inbred lines and advanced lines. The study aided to identify the tomato genotypes with superior nutritional, antioxidant and bioactive properties, that can be further utilized in tomato breeding program(s) aimed to improve these human health promoting traits in tomato fruit.

## Materials and methods

A diverse set of tomato germplasm with different genetic makeup including hybrids, parental lines and other lines were used for the estimation of bioactive pigments, antioxidant activities and nutritional parameters (**Table 1**). Different enzymatic and non-enzymatic antioxidants such as ascorbic acid (AsA), total flavonoids content (TFC), total phenolic content (TPC), ascorbate peroxidase (APX), superoxidase dismutase (SOD), catalase (CAT), peroxidase (POD), together with hydrolytic enzymes like alpha-amylase, protease and esterase activities were estimated. Important bioactive compounds like lycopene and total carotenoids, total chlorophyll, chlorophyll a, chlorophyll b and other biochemical parameter including Malondialdehyde (MDA) content, total soluble sugar (TSS), reducing sugars (RS), non-reducing sugars (NRS), total antioxidant capacity (TAC) and total antioxidant status (TOS) were also evaluated.

**Table 1:** Tomato genotypes used in the study.

Sr.#	Genotypes/accession #	Type	Origin/source	Other description/pedigree		Traits of importance
1.	NBH-149	D	NIAB, Pakistan	Hybrid	NCEBR-5 x AVTO1219	–
2.	NBH-150	D	NIAB, Pakistan	Hybrid	NCEBR-5 x AVTO1005	–
3.	NBH-151	D	NIAB, Pakistan	Hybrid	Naqeeb x AVTO1219	–
4.	NBH-152	D	NIAB, Pakistan	Hybrid	Naqeeb x AVTO1005	–
5.	NBH-154	D	NIAB, Pakistan	Hybrid	B-L-35 x AVTO1005	–
6.	NBH-182	D	NIAB, Pakistan	Hybrid	(Roma x LBR-7) x Flora-Dade	–
7.	NBH-188	D	NIAB, Pakistan	Hybrid	(Roma x LBR-10) x Flora-Dade	–
8.	NBH-190	D	NIAB, Pakistan	Hybrid	(Roma x LBR-17) x Flora-Dade	–
9.	NBH-196	D	NIAB, Pakistan	Hybrid	Canda-25 × AVTO1219	–
10.	NBH-200	D	NIAB, Pakistan	Hybrid	NCEBR-6 x AVTO1219	–
11.	NBH-204	D	NIAB, Pakistan	Hybrid	Galia x AVTO1219	–
12.	NBH-227	D	NIAB, Pakistan	Hybrid	B25 x AVTO1080	–
13.	NBH-228	D	NIAB, Pakistan	Hybrid	NCEBR-5 x AVTO1080	–
14.	NBH-229	D	NIAB, Pakistan	Hybrid	Naqeeb x AVTO1080	–
15.	NBH-235	D	NIAB, Pakistan	Hybrid	NCEBR-5 x B-31	–
16.	NBH-255	ID	NIAB, Pakistan	Hybrid	PRN x AVTO1003	–
17.	NBH-256	ID	NIAB, Pakistan	Hybrid	PRN x AVTO1005	–
18.	NBH-257	ID	NIAB, Pakistan	Hybrid	PRN x AVTO1080	–
19.	NBH-258	D	NIAB, Pakistan	Hybrid	B23 x AVTO1003	–
20.	NBH-259	D	NIAB, Pakistan	Hybrid	B23 x AVTO1005	–
21.	NBH-260	D	NIAB, Pakistan	Hybrid	B23 x AVTO1005	–
22.	NBH-261	D	NIAB, Pakistan	Hybrid	B24 x AVTO1003	–
23.	NBH-263	D	NIAB, Pakistan	Hybrid	B24 x AVTO1080	–
24.	NBH-265	D	NIAB, Pakistan	Hybrid	NCEBR-5 x AVTO1003	–
25.	NBH-266	D	NIAB, Pakistan	Hybrid	Galia x AVTO1003	–
26.	NBH-267	D	NIAB, Pakistan	Hybrid	Galia x AVTO1005	–
27.	NBH-268	D	NIAB, Pakistan	Hybrid	Riogrande x AVTO1003	–
28.	NBH-281	D	NIAB, Pakistan	Hybrid	Naqeeb x AVTO 1003	–
29.	NBH-282	D	NIAB, Pakistan	Hybrid	Naqeeb x H 24	–
30.	NBH-5	D	NIAB, Pakistan	Hybrid	B25 x NCEBR-6	–
31.	NBH-78	D	NIAB, Pakistan	Hybrid	Astra x Naqeeb	–
32.	NBH-95	D	NIAB, Pakistan	Hybrid	M-82 x Naqeeb	–
33.	Sundar F1	ID	ARRI, Pakistan	Hybrid	–	Tolerance against disease and temperature, tunnel crop, approved variety
34.	NIAB-Gohar	D	NIAB, Pakistan	Hybrid	(LBR-7 × Nagina)	High fruit firmness and yield, moderate disease tolerance, approved variety
35.	NIAB-Jauhar	D	NIAB, Pakistan	Hybrid	(Roma × LBR-10)	High fruit firmness and yield, moderate disease tolerance, approved variety
36.	Sahel F1	ID	Syngenta	Hybrid	–	Disease resistance, plum type
37.	T-1359 F1	D	Syngenta	Hybrid	–	Highly susceptible to blight
38.	Surkhail F1	ID	ARRI, Pakistan	Hybrid	–	High yield, fruit quality
39.	Iron-Lady F1	D	–	Hybrid	–	Disease resistance and fruit quality
40.	Ahmar F1	D	ARRI, Pakistan	Hybrid	–	Tolerance against disease and temperature, tunnel crop, approved variety
Parent genotypes						
Sr.#	Genotypes/Accession #	Type	Origin/Source	Other description/pedigree		Traits of importance
41.	AVTO1219 /CLN3241H-27	SD	AVRDC, Taiwan	Inbred line	Pedigree: CLN3241F1-34-28-2-20-5-28-27	Fair heat tolerance, disease resistance: late blight, tomato yellow leaf curl virus
42.	AVTO1003 /CLN3125L	D	AVRDC, Taiwan	Inbred line	*-*	Fair heat tolerance, resistance to diseases: tomato mosaic virus, tomato yellow leaf curl virus
43.	AVTO1005 /CLN3125P	D	AVRDC, Taiwan	Inbred line	Pedigree: CLN3125F2-21-4-13-1-0	Tomato yellow leaf curl resistance (TYLCD), Ty-1/Ty-3 and Ty-2
44.	AVTO1080/ CLN3022E (ck)	D	AVRDC, Taiwan	Inbred line	Pedigree: CLN3022F2-154-4-2-30-0	Tomato mosaic virus resistance (TMV), tomato yellow leaf curl virus susceptible
45.	B-31/Berika	D	Bulgaria			High yield
46.	B-23/Jaklin	D	Bulgaria			High yield
47.	B-24/Unkown line	D	Bulgaria			High yield
48.	B-25/Unknown line	D	Bulgaria			High yield
49.	Canada-25	D	EFUP			Fruit firmness, fruit quality and fruit size
50.	Flora-Dade/LA3242	D	University of Florida (1976)	OPV	*Solanum lycopersicum*	Heat tolerance, high yield, susceptible to early blight
51.	Galia	D	Gujranwala/Pakistan	OPV	*Solanum lycopersicum*	High yield, fruit quality
52.	H-24	D	ARRI, Pakistan	OPV	*Solanum lycopersicum*	High yield, fruit quality
53.	LBR-10/LB4	D	AVRDC, Taiwan		*Solanum lycopersicum*	Moderate early blight resistance,
54.	LBR-7/LB2	D	AVRDC, Taiwan		*Solanum lycopersicum*	Late/Early blight resistance, fruit size, fruit quality
55.	NCEBR-5 /LA3845	D	North Carolina, USA	Inbred line	*Solanum lycopersicum* Barksdale and Stoner (1977)	Late/Early blight resistance
56.	NCEBR-6 /LA3846	D	USA	Inbred line	*Solanum lycopersicum* Barksdale and Stoner (1978)	Early blight resistance
57.	M-82 /LA3475	D	TRGC, UC	Inbred line	*Solanum lycopersicum*	Cultivated tomato, fruit quality
58.	Nagina	D	Faisalabad, Pakistan	OPV	*Solanum lycopersicum*	High yield, fruit quality, disease resistance
59.	Naqeeb	D	Faisalabad, Pakistan	OPV	*Solanum lycopersicum*	High yield, good fruit size, excellent shelf life tolerant salinity and nickel stress
60.	PRN-28-10	ID	NIAB, Pakistan		*Solanum lycopersicum*	Fruit firmness
61.	Roma	D	USA, Mexico	OPV	*Solanum lycopersicum*	Plum shaped tomato, fruit quality and high yield
62.	Riogrande	D	USA/ARRI, Faisalabad	OPV	*Solanum lycopersicum*	Disease resistance: Fusarium 1 & 2, Verticillium wilt
63.	LA4157 /TA2893	D	TGRC, UC	BRIL	Solanum Lycopersicum × *Solanum Pimpinellifolium*	moderate resistance to early blight, fruit quality
64.	Astra	D	EFUP	OPV		
Lines						
Sr.#	Genotypes/Accession #	Type	Origin/Source	Other description/pedigree		Traits of importance
65.	17253	ID	ARRI, Pakistan	OPV		High yield, cherry tomato, disease resistance
66.	21354	D	Mexico			Fruit quality
67.	21396	D	Guatemala		*Solanum lycopersicum*	Disease resistance, high yield
68.	V-48 /PAK0010576	D	PGR N. Korea			
69.	AVTO 1009 /CLN3078G	D	AVRDC	Inbred line	Pedigree: CLN3078F1-12-6-25-8-4-0	Moderate heat tolerance, Disease resistance: Bacterial wilt, tomato mosaic virus (Tm22)
70.	AVTO 1010 /CLN3070J	D	AVRDC, Taiwan	Inbred line	—	
71.	AVTO 1311 /CLN3241R	SD	AVRDC, Taiwan	Inbred line	—	Moderate heat susceptible, Disease resistance: Late blight, bacterial wilt, fusarium wilt (R1 R2)
72.	AVTO 1315 /CLN3241Q	SD	AVRDC, Taiwan	Inbred line	Pedigree: CLN3241F1-34-28-2-20-10-17-27-25	Moderate heat susceptible, Disease resistance
73.	CLN2768A/LA1035	D	Ecuador/AVRDC	Wild type	*Solanum cheesmaniae*	Moderate resistance to late blight
74.	LA4097 /I.L. 12-1	D	Israel/TGRC, UC	IL	*Solanum Lycopersicum* × *Solanum Pennellie*	Drought tolerance, fruit quality
75.	LA4141 /TA2876	D	TGRC, UC	BRIL	*Solanum Lycopersicum* × *Solanum Pimpinellifolium*	Fruit quality, biotic and abiotic stress tolerance
76.	B-L-35/ LA4347	D	TRGC, UC	Inbred line	*Solanum lycopersicum*	Disease resistance, fruit quality
77.	LBR-17	D	AVRDC, Taiwan		*Solanum lycopersicum*	
78.	Lukullus/ LA0534	ID	Germany/TGRC	OPV	*Solanum lycopersicum*	Rabbit Resistant (roots and leaves are poisonous)
79.	Money maker/LA2706	ID	Bristol/ARRI, Pakistan	OPV	*Solanum lycopersicum*	Inexpensive compared to hybrid tomato, Blight prone, cold sensitive
80.	New cherry	ID	ARRI, Pakistan	Wild type		High yield, cherry type
81.	NI-Cherry	ID	ARRI, Pakistan	Wild type		High yield, cherry type
82.	V-83/011856	D				
83.	Pakit	ID	ARRI, Pakistan			Resistance to tomato fruit borer, high number of fruits per cluster
84.	Vendor/ LA3122	ID	TGRC, UC	OPV	*Solanum lycopersicum*	Tomato mosaic virus resistance
85.	West Virginia -63	ID	TGRC, UC	OPV	*Solanum lycopersicum*	Mild sweet flavor, late blight resistance (race T-0 and some resistance to T-1)
86.	UAF-1	ID	ARRI, Pakistan		Wild type	High yield, Cherry type
87.	LYP-1	D	ARRI, Pakistan		—	Fruit quality, high yield
88.	Nadir	D	ARRI, Pakistan	OPV	*Solanum lycopersicum*	High yield, good fruit size, excellent shelf life, sensitive to salinity and Nickel stress
Advance lines						
89.	CKD-6-15 F6	D	NIAB, Pakistan	AD		High fruit firmness
90.	CKD-8-15 F6	D	NIAB, Pakistan	AD		High fruit firmness
91.	MIL-10 (F4)	D	NIAB, Pakistan	AD	Riogrande x NCEBR-6	High fruit firmness
92.	MIL-13 (F4)	D	NIAB, Pakistan	AD	Naqeeb x NCEBR-6	High yielding line, high fruit firmness
93.	T-1359-6-15F6	D	NIAB, Pakistan	AD		High fruit firmness

NIAB, Nuclear Institute for Agriculture and Biology, Pakistan; AARI, Ayub Agricultural Research Institute, Faisalabad, Pakistan; NARC, National Agricultural Research Council, Pakistan; PARC, Pakistan Agricultural Research Council; TGRC, Tomato Genetic Resources Centre, United States of America; AVRDC, Asian Vegetable Research and Development Centre, Taiwan; EFUP, Establishment of facilitation unit for participatory vegetable seed and nursery production programme, Pakistan; PGR N. Korea, Plant Genetic Resource Center, North Korea; D, determinate; ID, Indeterminate; SD, Semi-determinate; OPV, Open Pollinated Variety; AD, Advance line; BRIL, Backcross Recombinant inbred; IL, Introgression Lines.

For fruit sample collection, field experiment was conducted during year 2018-2019 growing season, at Nuclear Institute for Agriculture and Biology (NIAB), Faisalabad, Pakistan. All standard agronomic practices were followed to keep tomato crop in good condition. Fully matured tomato fruits from each genotype were collected from the field in triplicates (May 2019) and stored at -80°C until further evaluation. The fruit compositional analysis was conducted at Plant Breeding and Genetic Division, Marker Assisted Breeding Lab-1, NIAB, Faisalabad.

## Estimation of antioxidant activities

### Tomato fruit sample extraction

Fruit sample (pericarp) weight (0.2 g) was taken out in 2 ml (50 mM) potassium phosphate buffer (pH 7.4). The supernatant was separated after centrifugation of 10 min at 14462 x g and 4°C. The extracted supernatant was used for the estimation of enzymatic and non-enzymatic (ascorbic acid, total flavonoid content, total phenolic compounds activities) by using different methods (Khalid and Hameed, 2017). A triplicated data of each genotype was collected for further investigations.

### Pigment estimation

The levels of pigments including lycopene, carotenoids, total chlorophyll, chlorophyll a and chlorophyll b, were estimated by a previously described method (Lichtenthaler and Wellburn, 1983). Tomato fruit sample weight 0.2 g was extracted in 80% acetone at -4°C centrifuged for 5 min at 10,000 × g using Sigma (Micro 1-14) centrifuge. The absorbance of chlorophyll a 663 nm, chlorophyll b 645 nm, lycopene, total carotenoids (480 nm) and total chlorophyll was measured at 663, 645, 505, 453 and 470 nm wavelength respectively using a spectrophotometer (SPH-003, HITACHI U-2800).

## Non-enzymatic antioxidants

### Ascorbic acid

A previously defined 2,6- dichloroindophenol (DCIP) method (Hameed et al., 2005) was followed to measure reduction in ascorbic acid (AsA) concentration. The reaction mixture contained 110 µl of DCIP (0.2 mg DCIP per ml of distilled water), 110 µl of 0.1% meta phosphoric acid, 100 µl sample extract and 900 µl distilled water. The absorbance of the reaction mixture was then measured at 520 nm using a spectrophotometer. Briefly, each molecule of ascorbic acid converts a molecule of DICP into DCIPH_2_ molecule. This conversion can be determined as a decline in absorbance at 520 nm by a spectrophotometer. A series of known ascorbic acid concentrations were used to prepare a standard curve. A simple regression equation was utilized to measure ascorbate concentrations in unspecified samples.

### Total flavonoid content

An aluminum chloride colorimetric method (Lin and Tang, 2007) was exploited to determine total flavonoid content (TFC). A reaction mixture containing tomato fruit sample (400 µl + 1.6 ml distilled water), 1 M potassium acetate (0.1 ml), 10% aluminum chloride hexahydrate (0.1 ml) and deionized water (2.8 ml) was prepared. The reaction mixture was then subjected to incubation at room temperature for 40-minutes, followed by measuring absorbance at 415 nm using a spectrophotometer. The standard curve was plotted using various known concentrations (0.005 to 0.1 mg/ml) of Rutin. The TFC was expressed as microgram per ml of the sample.

### Total phenolic contents

Total phenolic content (TPC) for each tomato genotype was determined by micro colorimetric technique (Ainsworth and Gillespie, 2007). Briefly, Folin-Ciocalteu (F-C) reagent was used for determining TPC in tomato fruit extract. For the purpose, 0.5 g of fruit sample was homogenized in 500 µl 95% methanol (ice-cold) using an ice-cold mortar and pestle. The samples were then incubated at room temperature in dark for 48 hours. When the incubation was completed, sample were centrifuged at 14,462 × g for 5 minutes at room temperature. The supernatant was removed and used for the measurement of TPC. The 100 µl of the supernatant was added with 100 ml of 10% (v/v) F-C reagent, vortex thoroughly, finally 800 μl of 700 mM Na2CO3 was added. Samples were then subjected to incubation at room temperature for an hour. Blank corrected absorbance of samples was measured at 765 nm. A standard curve was established using various known concentrations of gallic acid concentrations (300, 400, 500, 600, 700, and 800 mM/100 mL). The phenolic contents (gallic acid equivalents) of tomato samples were estimated using a linear regression equation.

## Enzymatic antioxidants

### Ascorbate peroxidase activity

Ascorbate peroxidase (APX) activity was estimated by homogenizing tomato fruit sample in 50 mM potassium phosphate buffer (pH 7), by exploiting previously established method (Dixit et al., 2001). By adding 200 mM potassium phosphate (pH 7.0), 0.5 M ethylenediamine tetra acetic acid (EDTA) and 10 mM ascorbic acid, an assay buffer was prepared. The buffer was then combined with 1 ml of H_2_O_2_ and 50 µl of supernatant. For estimation of APX activity, absorbance was recorded at 290 nm with 30 seconds interval. The decrease in absorbance indicated an ascorbic acid oxidation rate (Chen and Asada, 1989).

### Superoxide dismutase activity

Superoxide dismutase (SOD) activity was estimated by homogenizing tomato fruit samples in a medium consisting of 50 mM potassium phosphate buffer (pH 7.0), 0.1 mM EDTA, and 1 mM dithiothreitol (DTT) following previously reported method (Dixit et al., 2001). The SOD activity was determined by its ability to inhibit the photochemical reduction of nitro blue tetrazolium (NBT) following a previously described protocol (Giannopolitis and Ries, 1977). One unit of SOD activity was defined as the amount of enzyme that caused 50% inhibition of photochemical reduction of NBT.

### Catalase activity

Catalase (CAT) activity was determined by homogenizing fruit samples in a medium containing 50 mM potassium phosphate buffer (pH 7.0) and 1 mM dithiothreitol (DTT). CAT activity was determined by a previously defined method (Beers and Sizer, 1952). Estimation of CAT activity was carried out by preparing an assay buffer containing 50 mM phosphate buffer (pH 7.0), 59 mM H_2_O_2_ and 0.1 ml enzyme extract. At a wavelength of 240 nm the decline in absorbance of the reaction mixture was recorded after every 20 seconds for one min. One unit of CAT activity was defined as an absorbance change of 0.01 min^−1^. Enzyme activity was expressed on the bases of fruit weight.

### Peroxidase activity

Peroxidase (POD) activity was determined by homogenizing the fruit sample in a medium containing 50 mM potassium phosphate buffer (pH 7.0), 0.1 M EDTA, and 1 mM DTT. A previously described method (Chance and Maehly, 1957) with some modifications was used to measure POD activity. The assay solution for POD activity determination contained distilled water (535 μl), 250 µl of 200 mM phosphate buffer (pH 7.0), 100 µl of 200 mM guaiacol, 100 µl of 400 mM H_2_O_2_. Enzyme extract (15 µl) was added to initiate the reaction. After every 20 second the change in the absorbance was recorded at 470 nm for one min. An absorbance change of 0.01 min^−1^ was defined as one unit of POD activity. Enzyme activity was expressed on fruit weight bases.

## Hydrolytic enzymes

### Alpha-amylase activity

For the determination of fruit alpha-amylase activity, a previously defined method (Varavinit et al., 2002). Two reagents 3,5-dinitrosalicyclic acid (DNS) and 1% starch solution were used for the estimation of alpha-amylase activity. DNS reagent used for the assay was prepared by adding 96 mM DNS (1 g DNS in 50 ml of distilled water), 30 g of sodium potassium tartrate, 20 ml of 2 N NaOH and the final volume was made to 100 ml using distilled water. After mixing 0.2 ml sample + 1.8 ml distilled water and 1 ml of 1% starch solution, the reaction mixture was incubated for 3 min, then 1 ml of DNS reagent was added in each tube and placed in water bath for 15 min at 100°C. The boiled samples were then cooled at room temperature and finally 9 ml of distilled water was added. Absorption was observed at 540 nm using spectrophotometer.

### Protease activity

Fruit samples were extracted in 50 mM potassium phosphate buffer (pH 7.8) for protease activity estimation using Casein digestion assay (Drapeau, 1976). A reaction mixture containing 2 ml of 1% casein solution, 100 µl of enzyme extract and 2 ml of 10% TCA was prepared. The prepared reaction mixture was then filtered with a filter paper and absorbance was measured at 280 nm using spectrophotometer. For preparing 1% Casein solution, 1 g of casein, 50 ml of 0.01 N NaOH, 5 ml of 1 M Tris-base and 40 ml of distilled water was used, whereas the pH of the solution was maintained at 7.8 using phosphoric acid and final volume was made up to 100 ml. In this method, one unit is the quantity of an enzyme which delivers acid-soluble fragments equivalent to 0.001 A_280_ per min at 37°C with pH 7.8. Enzyme activity was expressed on a fruit weight basis.

### Esterase activity

A previously described method (Van Asperen, 1962) was exploited to determine α-esterases and β-esterases activity by using α-naphthyl acetate and β-naphthyl acetate substrates, respectively. The reaction mixture was composed of substrate solution (0.04 M phosphate buffer (pH 7), 1% acetone, and 30 mM α or β-naphthyl acetate) along with the enzyme extract. The mixture was incubated in dark for exactly 15 min at 27°C. A staining solution (1% Fast blue BB and 5% SDS combined in a ratio of 2:5) was mixed with the above-mentioned reaction mixture and incubated for another 20 min in dark at 27°C. The quantity of α- and β-naphthol produced was estimated by measuring the absorbance at 590 nm. Enzyme activity was α or β naphthol produced in μM min^−1^ per g fruit weight, using a standard curve.

## Other biochemical assays

### Total soluble sugars

The phenol–sulphuric acid reagent method (Dubois et al., 1951) was exploited for the estimation of total sugar content. The reaction mixture contained sample extract, reagent 1 (5% phenol solution) and reagent 2 (96% sulphuric acid). After adding 250 µl of reagent 1, 1.25 ml of reagent 2 and 500 µl of sample extract, the reaction mixture was placed in a water bath for 20 minutes at 30°C, later absorbance of the reaction mixture was measured at 490 nm using a spectrophotometer.

### Reducing sugars

For the determination of fruit reducing sugars content dinitrosalicylic acid (DNS) method (Miller 1959) was used. The assay mixture was composed of 200 µl of sample extract, 1 ml of DNS reagent and 1.8 ml of distilled water. After adding the above-mentioned reagents with sample extract, the reaction mixture was heated in water bath for 15 minutes at 100°C, then the boiled reaction mixture was allowed to cool at room temperature and 9 ml of distilled water was added in each test tube. The absorbance of the reaction mixture was finally measured at 540 nm by using a spectrophotometer. DNS reagent used for the assay was prepared by adding 96 mM DNS (1 g DNS in 50 ml of distilled water), 30 g of sodium potassium tartrate, 20 ml of 2 N NaOH and the final volume was made to 100 ml using distilled water. Non-reducing sugars were estimated by the difference in total soluble sugars and reducing sugars.

### Malondialdehyde content

Malondialdehyde (MDA), a byproduct of lipid peroxidation was estimated by the thiobarbituric acid (TBA) reaction method (Heath and Packer, 1968), with minor changes (Dhindsa et al., 1981; Zhang and Kirkham, 1994). A fruit sample weight of 0.25 g was homogenized in 5ml TCA (0.1%). The homogenate was then centrifuged for about 5 min at 10,000 × g. In 1 ml of aliquot of supernatant, 4 ml TCA (20%) containing 0.5% TBA were added. The mixture was then heated for 30 min at 95°C and then immediately cooled in an ice bath. A centrifugation of 10,000 × g for 10 min was done. The absorbance of the supernatant at 532nm was measured and the value for non-specific absorption at 600nm was subtracted. MDA content was measured by using extinction coefficient of 155 mM^−1^ cm^−1^.

### Total oxidant status

For the estimation of total oxidant status (TOS) a previously used method (Erel, 2005) based upon the oxidation of ferrous ion to ferric ion by oxidants present in the sample in an acidic medium and the measurement of ferric ion by xylenol orange (Harma et al., 2005) was used. The assay mixture contained reagent one (R1), reagent two (R2), along with the sample extract. The reagent R1 was the stock xylene orange solution containing 75 µl xylenol orange dye (0.38 g xylenol orange in 500 µl of 25 mM H_2_SO_4_), 0.409 g of NaCl, 500 µl of glycerol and final volume was made up to 50 ml with 25 mM H_2_SO_4_. The reagent 2 (R2) contained 0.0317 g of o-dianisidine and 0.0196 g of ferrous ammonium sulfate in 10 ml of 25 mM H_2_SO_4_. After adding 900 µl of reagent 1, 140 µl of sample and 44 µl of reagent 2 and the reaction mixture was incubated for 5 minutes. Then the absorbance of the reaction mixture was measured at 560 nm by using a spectrophotometer. A standard curve was formed using known concentrations of H_2_O_2._ The results were explained in μ M H_2_O_2_ equivalent per L.

### Total antioxidant capacity

Total antioxidant capacity was estimated by a previously reported method (Erel, 2004). The 2,2-Azinobis-3-ethylbenzthiazolin-6-sulfonic acid (ABTS) assay exhibits a decline of 2,2-azino-bis (3 ethylbenzothiazoline-6- sulfonate) radical cation ABTS+ (blue green in colour) into the actual ABTS (colorless compound), representing the presence of antioxidant in the tested sample. The antioxidant content present in the sample decolorizes the ABTS+ radical cation. The reaction mixture for TAC estimation contained sample extract, reagent R1 and reagent R2. Reagent 1 contained 94 ml of 0.4 M sodium acetate and 6 ml of 0.4 M glacial acetic acid, the pH of reagent 1 was maintained at 5.8. The reagent 2 contained 0.75 ml of 30 mM sodium acetate and 9.25 ml of glacial acetic acid. Then, 3.52 µl was taken out from R2 and 3.52 µl of 35% of hydrogen peroxide solution was added in R2. Finally, 10 mM 2,2-Azinobis-3-ethylbenzthiazolin-6-sulfonic acid (ABTS) (0.549 g in 10 ml H_2_O_2_) was added in in above-mentioned solution. Assay was performed by adding 1ml of reagent 1, 25 µl sample extract and 100 µl reagent 2 incubated for 5 minutes and absorbance was measured at 660 nm using spectrophotometer. Ascorbic acid was used as a standard to develop standard curves. The range of concentrations for ascorbic acid was between 0.075 and 2.0 mM/L. The amount of antioxidant present in the sample was expressed as μM of AsA equivalent to 1 g.

## Statistical analysis

For Statistical analysis the computer software Microsoft Excel along with XLSTAT (Version 2021.3.01), (http://www.xlstat.com) was used. Descriptive statistics were applied to organize the data. The data was expressed in mean ± SD. Analysis of variance (ANOVA) was performed for the data using three replications. The significance level of the data was tested by analysis of variance and Tukey (HSD) test at P < 0.05 using software XLSTAT. Principal component analysis and Pearson correlation test was performed.

## Results

Tomato genotypes including hybrids, parents and other lines were divided into three categories low, medium and high based upon the variability in their mean values for different tested parameters (**Table 2**). Detailed description of the results is as follows.

**Table 2:** Scale for categorization of tomato genotypes having low, medium, and high values of different biochemical parameters.

Parameters		Range of values		Number of genotypes per category			
				Hybrids(Total 40)	Parents(Total 24)	Lines(Total 24)	Advance lines(Total 5)
1. Pigments	a. Lycopene	Low	1.5-2.5 (mg/100g FW)	14	7	8	2
		Medium	2.5-5.5 (mg/100g FW)	21	15	15	3
		High	5.5-7.9 (mg/100g FW)	5	2	1	–
	b. Total carotenoids	Low	2-5 (mg/100g FW)	12	3	3	1
		Medium	5-10 (mg/100g FW)	23	18	19	4
		High	10-18 (mg/100g FW)	5	3	2	–
	c. Total chlorophyll	Low	60-100 (µg/100g FW)	26	14	16	2
		Medium	100-160 (µg/100g FW)	11	8	7	3
		High	160-215 (µg/100g FW)	3	2	1	–
	d. Chlorophyll a	Low	24-40 (µg/100g FW)	15	8	4	–
		Medium	40-70 (µg/100g FW)	20	10	15	2
		High	70-100 (µg/100g FW)	5	6	5	3
	e. Chlorophyll b	Low	10-40 (µg/100g FW)	14	5	8	2
		Medium	40-70 (µg/100g FW)	20	15	14	3
		High	70-120 (µg/100g FW)	6	4	2	–
2. Non enzymatic antioxidants	a. Ascorbic acid	Low	100-350 (µg/g FW)	7	5	6	2
		Medium	350-380 (µg/g FW)	27	17	15	1
		High	380-450 (µg/g FW)	6	2	3	2
	b. Total flavonoid content	Low	1,000-1,500 (µg/100g FW)	27	17	14	3
		Medium	1,500-3,000 (µg/100g FW)	7	5	5	2
		High	3000-5,000 (µg/100g FW)	6	2	5	–
	c. Total phenolic compounds	Low	300-1000 (µM/g FW)	4	4	–	–
		Medium	1000-10,000 (µM/g FW)	35	19	19	5
		High	10,000-14,650 (µM/g FW)	1	1	5	–
3. Enzymatic antioxidants	a. Ascorbate peroxidase	Low	200-600 (U/g FW)	17	7	12	2
		Medium	600-1,000 (U/g FW)	15	14	10	3
		High	1000-2,000 (U/g FW)	8	3	2	–
	b. Superoxide dismutase	Low	20-150 (U/g FW)	20	12	10	3
		Medium	150-250 (U/g FW)	18	11	11	2
		High	250-350 (U/g FW)	2	1	3	–
	c. Catalase	Low	100-300 (U/g FW)	2	2	–	–
		Medium	300-700 (U/g FW)	29	16	15	4
		High	700-1,200 (U/g FW)	9	6	9	1
	d. Peroxidase	Low	150-1,000 (U/g FW)	24	13	15	4
		Medium	1,000-5,000 (U/g FW)	14	7	8	1
		High	5,000-8,000 (U/g FW)	2	4	1	–
4. Hydrolytic enzymes	a. Alpha-amylase	Low	16-100 (mg/g FW)	9	6	6	–
		Medium	100-200 (mg/g FW)	26	14	12	5
		High	200-245 (mg/g FW)	5	4	6	–
	b. Protease	Low	5,000-6,500 (U/g FW)	22	12	11	1
		Medium	6,500-7,500 (U/g FW)	16	10	12	4
		High	7,500-8,500 (U/g FW)	2	2	1	–
	c. Esterase	Low	10-25 (µM/min/g FW)	17	8	7	1
		Medium	25-35 (µM/min/g FW)	14	13	11	2
		High	35-50 (µM/min/g FW)	9	3	6	2
5. Sugars	a. Total soluble sugars	Low	20-35 (mg/g FW)	5	4	7	–
		Medium	35-60 (mg/g FW)	26	16	10	4
		High	60-80 (mg/g FW)	9	4	7	1
	b. Reducing sugars	Low	21-40 (mg/g FW)	27	17	14	3
		Medium	40-60 (mg/g FW)	7	5	5	2
		High	60-71 (mg/g FW)	6	2	5	–
	c. Non-reducing sugar	Low	1-5 (mg/g FW)	12	8	6	
		Medium	5-14.4 (mg/g FW)	18	11	15	3
		High	14.4-20 (mg/g FW)	10	5	3	2
6. Other biochemical parameter	a. Malondialdehyde content	Low	20-80 (µM/g FW)	24	12	14	2
		Medium	80-150 (µM/g FW)	10	11	5	3
		High	150-270 (µM/g FW)	6	1	5	–
	b. Total oxidant status	Low	200-3,000 (µM/g FW)	19	9	7	4
		Medium	3,000-10,000 (µM/g FW)	11	8	8	–
		High	10,000-17,000 (µM/g FW)	10	7	9	1
	c. Total antioxidant capacity	Low	1-7 (µM/g FW)	14	–	4	2
		Medium	7-11 (µM/g FW)	23	17	17	3
		High	11-12 (µM/g FW)	3	7	3	–

## Pigment analysis

### Lycopene content

**Figure 1:**
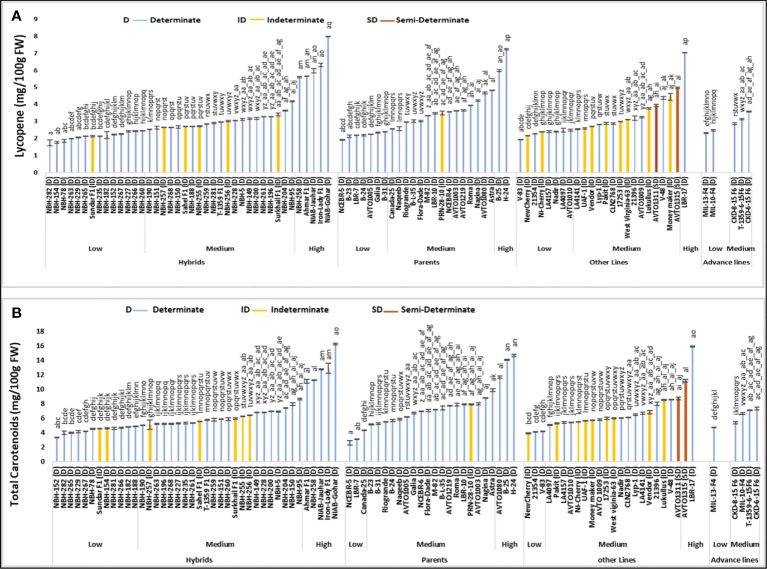
Comparison of fruit **(A)** lycopene and **(B)** total carotenoids content in different tomato genotypes (mean value ± SD). Mean value with varying alphabet differs significantly (p < 0.05, Tukey’s HSD).

Among forty hybrid tomato genotypes tested for their fruit lycopene content, fourteen exhibited low mean values ranging from 1.74 to 2.45 mg/100 g FW (**Figure 1**). For determinate tomato genotype, a local hybrid tomato NBH-282 showed the lowest mean value (1.74 mg/100 g FW) for fruit lycopene content. Twenty-one hybrids exhibited intermediate values for fruit lycopene content ranging from 2.53 to 4.17 mg/100 g FW. Five hybrids were grouped in the high category for fruit lycopene content with the mean values ranging from 5.58 to 7.96 mg/100 g FW. The highest value for determinate tomato genotype was observed in a local hybrid variety NIAB-Gohar (7.96 mg/100 g FW).

Out of twenty-four tomato parent genotypes tested for fruit lycopene content, seven were grouped into low category with their mean values ranging from 1.90 to 2.37 mg/100 g FW. The lowest value for determinate tomato parent genotype was observed in NCEBR-5 (1.90 mg/100 g FW). Fifteen parents showed intermediate fruit lycopene mean values ranging from 2.56 to 4.82 mg/100 g FW. Only two parent tomato genotypes were grouped into the high category for fruit lycopene content, with the highest value observed in a determinate parent line H-24 (7.23 mg/100 g FW).

Total twenty-four tomato lines tested for fruit lycopene content, eight were categorized into low category with their mean values ranging from 1.93 to 2.49 mg/100 g FW. The lowest value was detected in a determinate tomato line V-83 (1.93 mg/100 g FW). Fifteen tomato lines were placed in the medium category for fruit lycopene content ranging from 2.53 to 4.95 (mg/100 g FW). The highest value for fruit lycopene content was observed in determinate tomato line, LBR-17 (7.02 mg/100 g FW).

Total five tomato advance lines tested for fruit lycopene content, two were grouped into a low category with the lowest value detected in an advance line, MIL-13-F4 (2.29 mg/100 g FW). Three advance lines exhibited medium values for fruit lycopene content ranging from 2.85 to 3.57 (mg/100 g FW).

Total sixteen indeterminate tomato genotypes were tested for fruit lycopene content, with the lowest mean value observed in a local hybrid Sundar F1 (2.13 mg/100 g FW), and the highest mean value observed in an indeterminate variety Moneymaker (4.43 mg/100 g FW). Two semi-determinate tomato lines under study, exhibited intermediate values for fruit lycopene content. Semi-determinate line, AVTO1311 showed a mean value of 3.92 mg/100 g FW, and semi-determinate line AVTO1315 showed a value of 4.95 mg/100 g FW respectively.

### Total carotenoids

The data of mean values for tomato fruit total carotenoid content showed that twelve hybrids out of a total twenty-four fall under the low category with a mean value ranging from 3.35 to 4.86 mg/100 g FW (**Figure 1**). The lowest mean value for determinate tomato type was observed in a local hybrid, NBH-152 (3.35 mg/100 g FW). In the intermediate category, twenty-three tomato hybrids were grouped with the total fruit carotenoids content ranging from 5.02 to 8.62 mg/100 g FW. Five tomato hybrids were placed in the high category for fruit total carotenoid content with their mean values ranging from 11.07 to 16.23 mg/100 g FW. The highest mean value for determinate tomato genotypes was observed in a local hybrid variety, NIAB-Gohar (16.23 mg/100 g FW).

Among twenty-four parent tomato genotypes, three were grouped into the low category for fruit total carotenoid content ranging from 2.56 to 4.35 mg/100g FW. The lowest value for determinate tomato genotype was observed in a parent, NCEBR-5 (2.56 mg/100 g FW). Eighteen parent tomato genotypes were categorized in medium category with their mean values varying from 5.11 to 9.82 mg/100 g FW. In the high category for fruit total carotenoid content, three parent tomato genotypes were grouped with the mean values ranging from 11.65 to 14.63 mg/100 g FW, while the highest mean value for determinate tomato genotype was observed in a parent, H-24 (14.6 mg/100 g FW).

Twenty-four tomato lines were tested for fruit total carotenoid content, three were grouped into low category ranging from 3.90 to 4.19 mg/100 g FW. The lowest value for determinate tomatoes was observed in an exotic line 21354 (4.12 mg/100 g FW). In the intermediate category nineteen lines were grouped with their fruit total carotenoid content ranging from 5.06 to 8.69 mg/100 g FW. Two lines were included in the high category for fruit total carotenoids content, the highest value was observed in a determinate line LBR-17 (15.88 mg/100 g FW).

Tomato advance lines showed low and medium values for fruit total carotenoids content. The lowest value was observed in a determinate advance line MIL-13-F4 (4.68 mg/100 g FW). Four advance lines were placed in an intermediate category for fruit total carotenoids content ranging from 5.35 to 7.31 mg/100 g FW, respectively.

Among sixteen indeterminate genotypes tested, the lowest value for fruit total carotenoid content was observed in an indeterminate cherry tomato NewCherry (3.90 mg/100g FW), while the highest mean value was observed in an indeterminate German variety Lukullus (8.48 mg/100 g FW). The two semi-determinate inbred lines AVTO1311 and AVTO1315 showed a mean value of 8.69 and 11.14 mg/100 g FW for fruit total carotenoid content.

### Total chlorophyll content

**Figure 2:**
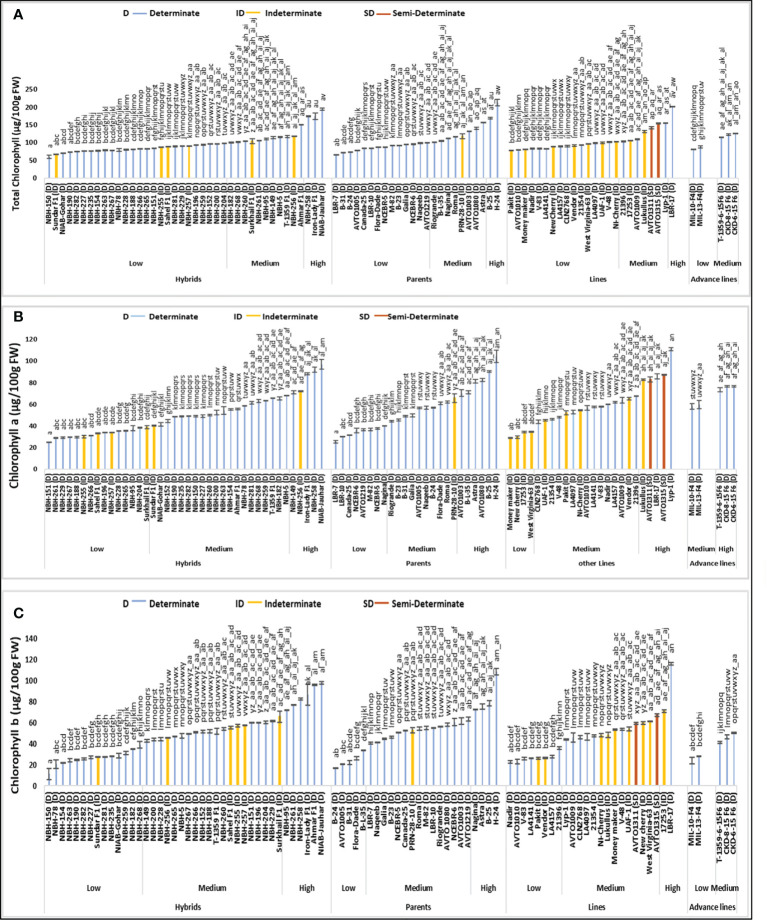
Comparison of fruit **(A)** total chlorophyll content, **(B)** chlorophyll a and **(C)** chlorophyll b in different tomato genotypes (mean value ± SD). Mean value with varying alphabet differs significantly (p < 0.05, Tukey**’**s HSD).

Among forty tomato hybrid genotypes, total twenty-six were placed in the low category for fruit total chlorophyll content, with their mean values ranging from 60.09 to 98.35 µg/100 g FW (**Figure 2**). The lowest value for determinate tomato genotype was observed in a hybrid NBH-150 (60.09 µg/100 g FW). In the intermediate category, eleven tomato hybrids were placed with their mean values ranging from 100.40 to 151.67 (µg/100 g FW). Whereas in the high category for fruit total chlorophyll content three hybrids were placed with their mean values ranging from 174.28 to 194.74 µg/100 g FW. While the highest value for determinate tomato was observed in a local hybrid, NIAB-Jauhar (194.74 µg/100 g FW).

Tomato parent genotypes evaluated for their fruit total chlorophyll content showed significant variation in the mean values. Fourteen parent genotypes out of a total twenty-four were categorized into low category with their mean values ranging from 65.73 to 99.67 (µg/100 g FW). The lowest value for determinate tomatoes was observed in a parent line, LBR-7 (65.73 µg/100 g FW). In the intermediate category eight parents were grouped with their mean values ranging from 100.59 to 156.64 (µg/100 g FW). The high category for tomato fruit total chlorophyll content contained two determinate parent genotypes, B-25 (168.76 µg/100 g FW) and H-24 (212.67 µg/100 g FW).

Tomato lines tested for their fruit total chlorophyll content showed significant variation. Sixteen out of total twenty-four lines were grouped into low category for fruit total chlorophyll content with their mean values ranging from 78.22 to 102.38 (µg/100 g FW). The lowest value of total chlorophyll content for determinate tomato lines was observe in an exotic line, AVTO1010 (79.59 µg/100 g FW). Seven lines were grouped into the medium category for total chlorophyll content in fruit, with a range of 103.39 to 155.26 µg/100 g FW. In the high category only one line (LBR-17) was placed with a mean value of 201.82 µg/100 g FW, respectively.

Tomato advance lines were grouped into the low and medium category for fruit total chlorophyll content, based upon the observed variation in the mean value. In the low category two tomato advance lines were placed with the lowest value observed in MIL-10-F4 (81.98 µg/100 g FW). Three tomato advance lines were placed in the medium category for fruit total chlorophyll content with their mean value ranging from 114.52 to 126.77 µg/100 g FW, respectively.

Among sixteen indeterminate tomato genotypes, the lowest fruit total chlorophyl content was observed in a local hybrid, Sundar F1 (67.85 µg/100 g FW). While the highest mean value for total chlorophyll content was observed in an indeterminate variety, Lukullus (131.20 µg/100 g FW), respectively. The two semi-determinate lines tested showed medium mean values for fruit total chlorophyll content, AVTO1311 showed a value of 142.39 µg/100 g FW, while AVTO1315 exhibited a value of 154.21 µg/100 g FW, respectively.

### Chlorophyll a content

Based upon the variation in the mean values for fruit chlorophyll a content, out of a total of twenty-four hybrid tomato genotypes fifteen hybrids were categorized into low category with their mean values ranging from 24.79 to 38.71 µg/100 g FW (**Figure 2**). The lowest value for determinate tomatoes was observed in a local hybrid, NBH-151 (24.79 µg/100 g FW). In the intermediate category for chlorophyll a content twenty hybrid tomatoes were grouped, with their mean values ranging from 40.21 to 67.96 µg/100 g FW. Category with the highest value for chlorophyll a content contained five tomato hybrids, with their mean values varying from 71.22 to 96.93 µg/100g FW. The highest chlorophyll a content for determinate tomatoes was observed in a local hybrid variety, NIAB Jauhar (96.93 µg/100 g FW).

Among twenty-four tomato parents, eight were grouped in the low category for fruit chlorophyll a content, with their mean values ranging from 25.31 to 39.87 µg/100g FW. The lowest value for determinate tomatoes was observed in a parent line, LBR-7 (25.31 µg/100 g FW). In the medium category ten parents were placed, with the values ranging from 44.26 to 65.70 µg/100 g FW. Six tested parent genotypes were included in the high category for chlorophyll a content ranging from 70.35 to 104.19 µg/100 g FW. The highest chlorophyll a content of 104.19 µg/100 g FW was observed in a determinate parent tomato, H-24 respectively.

Out of twenty-four tomato lines tested, four fall under the low category for fruit chlorophyll a content ranging from 28.79 to 34.17 µg/100 g FW. In the intermediate category fifteen lines were grouped with mean values ranging from 43.83 to 67.75 µg/100 g FW. In high category for chlorophyll a content, five tested lines were categorized with mean value ranging from 82.77 to 111.10 µg/100 g FW. The highest value for determinate tomatoes was observed in a local line Lyp-1 (111.10 µg/100 g FW).

Tomato advance lines showed medium and high values for fruit chlorophyll a content. Based upon the detected variation two advance lines MIL-10-F4 and MIL-13-F4 were grouped into an intermediate category with the mean values of 58.12 and 59.68 (µg/100g FW) for fruit chlorophyll a content. The remaining tomato advance lines showed high values for fruit chlorophyll a content, ranging from 73.43 to 76.53 µg/100g FW, respectively.

Among sixteen indeterminate tomato genotypes tested for fruit chlorophyll a content, the lowest value was observed in an indeterminate genotype Moneymaker (28.79 µg/100 g FW), while the highest value of chlorophyll a content was observed in an exotic indeterminate variety, Lukullus (82.77 µg/100 g FW). Semi-determinate inbred lines AVTO1311 and AVTO1315 were grouped in high category for fruit chlorophyll a content, with the mean values of 83.09 and 87.06 (µg/100g FW), respectively.

### Chlorophyll b content

Total forty tomato hybrids tested for fruit chlorophyll b content, fourteen were placed in the low category ranging from 11.13 to 38.86 µg/100 g FW (**Figure 2**). The lowest value for determinate tomato was observed in local hybrid, NBH-150 (11.13 µg/100 g FW). In the intermediate category twenty tomato hybrids were grouped with the mean values ranging from 42.94 to 65.63 µg/100 g FW. Six hybrid tomatoes were grouped into high category, with their mean value ranging from 72.07 to 97.80 µg/100 g FW. The highest value for determinate tomato was observed in a local hybrid NBH-Jauhar (97.80 µg/100 g FW).

Twenty-four tomato parent genotypes tested showed significant variation in their fruit chlorophyll b content. In the low category five tomato parents were placed, ranging from 16.92 to 34.03 µg/100 g FW. The lowest value for determinate tomato was observed in parent line B-24 (16.92 µg/100 g FW). In the intermediate category for fruit chlorophyll b content fifteen parents were grouped, with their mean values ranging from 40.41 to 63.41 µg/100 g FW. The category with the highest mean values for chlorophyll b content consisted of four parents ranging from 72.93 to 108.47 µg/100 g FW. The highest value for determinate tomato was observed in a parent, H-24 (108.47 µg/100g FW).

Tomato lines exhibited significant variation for fruit chlorophyll b content. Out of a total twenty-four lines tested, eight lines were categorized into low category, with their mean values ranging from 22.91 to 35.64 (µg/100 g FW). The lowest value for determinate tomato was observed in a line, Nadir (22.91 µg/100 g FW). In the intermediate category for fruit chlorophyll b content fourteen lines were grouped, with their mean values ranging from 44.16 to 67.15 (µg/100 g FW). In the high category two tomato lines were placed with their mean values ranging from 70.91 to 116.07 (µg/100 g FW). Highest chlorophyll b content for determinate tomato was observed in a line LBR-17 (116.07 µg/100 g FW).

Total five tomato advance lines were tested for fruit chlorophyll b content; low and medium values were detected among these lines. In the low category two advance lines were placed, the lowest value was observed in an advance line MIL-10-F4 (23.86 µg/100 g FW). In the medium category three advance lines were grouped, with their mean values ranging from 41.08 to 50.24 µg/100 g FW.

Among the indeterminate tomato genotypes lowest value for fruit chlorophyll b content was observed in Pakit (26.18 µg/100 g FW), while the highest value was observed in an indeterminate genotype, 17253 (70.91 µg/100 g FW). The two semi- determinate lines showed a medium value for chlorophyll b content, with the mean values of 59.30 µg/100 g FW (AVTO1311) and 67.15 µg/100 g FW, (AVTO1315) respectively.

## Non-enzymatic antioxidants

### Ascorbic acid

**Figure 3:**
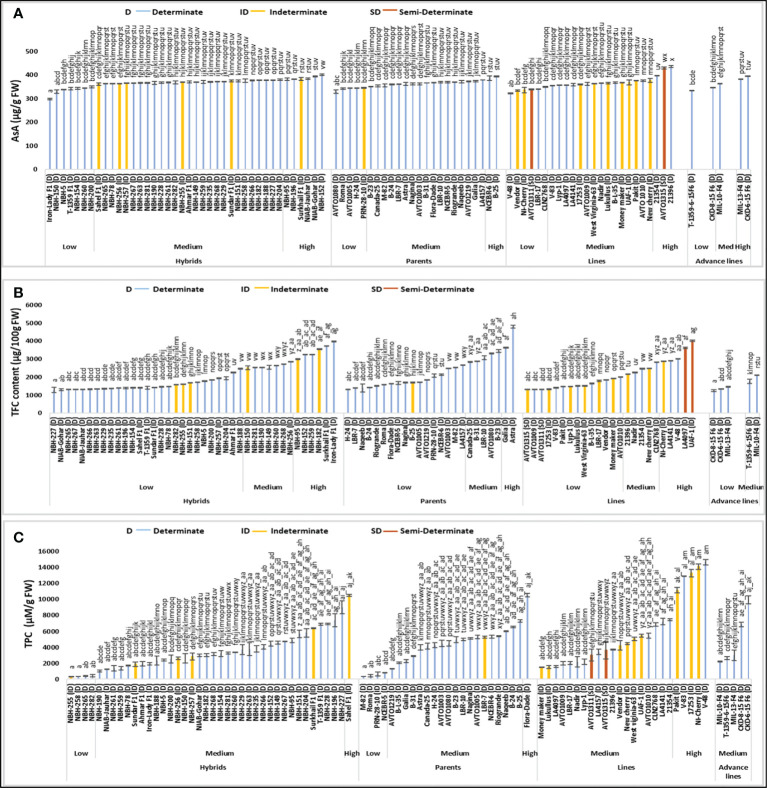
Comparison of fruit ascorbic acid (AsA) **(A)**, total flavonoid content (TFC) **(B)** and total phenolic compounds (TPC) **(C)** in different tomato genotypes (mean value ± SD). Mean value with varying alphabet differs significantly (p < 0.05, Tukey**’**s HSD).

Total forty tomato hybrids tested for fruit ascorbic acid (AsA) content showed significant variation. Seven tomato hybrids were grouped into low category with their mean values ranging from 298.00 to 349.25 µg/g FW (**Figure 3**). The lowest fruit ascorbic acid content for determinate tomato was detected in an exotic hybrid, Iron Lady F1 (298.00 µg/g FW). In the intermediate category, twenty-seven hybrids were grouped with the mean values varying between 361.50 to 379.50 µg/g FW. Total six tomato hybrids were grouped into high category, with their values ranging from 381.25 to 400.75 µg/g FW. The highest value for determinate tomato was observed in hybrid, NBH-152 (400.75 µg/g FW) respectively.

Tomato parents showed significant variation in the mean values for fruit ascorbic acid content. Five out of total twenty-four parents were grouped into the low category, with the mean values ranging from 329.50 to 345.00 µg/g FW. The lowest value for determinate tomato was observed in an AVRDC line, AVTO1080 (329.50 µg/g FW). In the medium category seventeen parents were placed with their mean values ranging from 350.00 to 379.25 µg/g FW. Two determinate tomato parents, NCEBR-6 (386.00 µg/g FW) and B-25 (394.25 µg/g FW) showed high values for fruit ascorbic acid content.

Out of twenty-four tomato lines tested, six exhibited comparatively low values for fruit ascorbic acid content. The mean values for tomato lines placed in low category varied between 321.75 to 349.50 µg/g FW. While the lowest value for determinate tomato was observed in a line, V-48 (321.00 µg/g FW). In the intermediate category fifteen lines were grouped with the mean values varying from 355.00 to 376.50 µg/g FW. Three lines exhibited high values for fruit ascorbic acidic content, ranging from 396.76 to 435.25 µg/g FW. The highest value for determinate tomato was observed in a line 21396 (435.25 µg/g FW) respectively.

Comparative analysis of the advance lines exhibited low, medium and high values for fruit ascorbic acidic content. Two advance lines showed low values while the lowest was observed in an advance line, T-1359-6-15 F6 (332.75 µg/g FW). MIL-10-F4 showed a medium value for ascorbic acid content (363.75 µg/g FW), while MIL-13-F4 (381.25 µg/g FW) and CDK-6-15 F6 (394.50 µg/g FW) showed high value for fruit ascorbic acid content.

A total of sixteen indeterminate tomato lines were tested, the lowest value for ascorbic acid was observed in an indeterminate line, Vendor (334.00 µg/g FW). The highest value was observed in indeterminate hybrid tomato, Surkhail F1 (383.75 µg/g FW). The two semi-determinate lines showed low and high values for fruit ascorbic acid content. AVTO1311 (339.25 µg/g FW) was placed in the low category whereas, AVTO1315 (429.25 µg/g FW) was placed in high category.

### Total flavonoid content

Significant variation was observed among forty tested tomato hybrids for fruit total flavonoid content (TFC). Twenty-seven hybrids were categorized in low category, ranging from 1295.33 to 2466.74 µg/100 g FW (**Figure 3**). The lowest value for determinate tomato was observed in a hybrid, NBH-227 (1295.335 µg/100 g FW). Seven hybrids were placed in the medium category with their mean values ranging from 2525.05 to 2858.98 µg/100 g FW. In the high category for total flavonoid content six hybrids were grouped, with their mean values varying between 2986.19 to 3972.08 (µg/100 g FW) and the highest value for determinate tomato was observed in a hybrid, Iron lady F1 (3972.08 µg/100 g FW).

Twenty-four tomato parents tested for fruit total flavonoid content exhibited significant variation. Seventeen parents were placed in the low category with their mean values ranging from 1321.83 to 2662.86 µg/100 g FW. For determinate tomato lowest value was observed in a parent, H-24 (1321.83 µg/100 g FW). Five parents were placed in the medium category for fruit total flavonoid content, ranging from 2837.78 to 3447.33 (µg/100 g FW). In the high category, two determinate tomatoes, Galia (3643.45 µg/100 g FW) and Astra (4793.66 µg/100 g FW) were placed.

Twenty-four tomato lines evaluated for the fruit total flavonoid content varied significantly, while fourteen lines were placed in a low category, ranging from 1321.83 to 2000.30 µg/100 g FW. The lowest value for determinate tomato was observed in a line, AVTO1009 (1321.83 µg/100 g FW). Five tested lines showed intermediate value for total flavonoid content, ranging from 2164.61 to 2832.48 µg/100 g FW. Another five lines showed high mean values for total flavonoid content, ranging from 2885.48 to 4009.19 µg/100 g FW. The highest value for determinate tomato was observed in an exotic line, LA4097 (3627.55 µg/100 g FW).

Tomato advance lines showed low and medium value for fruit total flavonoid content. Three advance lines were included in the low category, ranging from 1242.33 to 1459.65 µg/100 g FW. The lowest value was observed in an advance line, CKD-8-15-F6 (1242.33 µg/100 g FW). Two tested tomato advance lines were placed in the medium category, T-1359-6-15 F6 (1767.08 µg/100 g FW) and MIL-10-F4 (2106.31 µg/100 g FW).

Among the indeterminate tomato genotypes, the lowest value for total fruit flavonoid content was observed in 17253 (1332.43 µg/100 g FW) and the highest value was observed in an indeterminate line, UAF-1 (4009.19 µg/100 g FW) respectively. The two semi-determinate tomato lines tested showed low values for fruit total flavonoid content, whereas lowest mean was observed in AVTO1315 (1321.83 µg/100 g FW) followed by AVTO1311 (1332.43 µg/100 g FW).

### Total phenolic compounds

Significant variation was observed for fruit total phenolic compounds (TPC) among forty tested tomato hybrids. In the low category four hybrids were placed with their mean values ranging from 325 to 450 µM/g FW (**Figure 3**). The lowest value for determinate tomato was observed in a hybrid, NBH-258 (350 µM/g FW). In the intermediate category thirty-five tested hybrids were grouped, with their mean values ranging from 1025 to 9000 µM/g FW, respectively. Sahel F1 was the only hybrid tomato grouped in the high category for fruit total phenolic compounds with the mean values 10500 µM/g FW.

Twenty-four tomato parents tested for fruit total phenolic compound showed significant variation. In the low category four parent genotypes were categorized ranging from 350 to 800 µM/g FW, with the lowest mean value for determinate tomato observed in a parent, M-82 (350 µM/g FW). In the intermediate category for fruit total phenolic compounds, nineteen parents were categorized, with the mean values ranging from 1300 to 7300 µM/g FW. The highest value of fruit total phenolic compound for determinate tomato was observed in the parent, Flora-Dade (10525 µM/g FW) respectively.

Twenty-four tomato lines evaluated for fruit total phenolic compound were categorized into medium and high category. In the intermediate category nineteen lines were placed with their mean values ranging from 1475 to 7475 µM/g FW. In the high category five lines were placed, ranging from 11150 to 14650 µM/g FW. The highest value for determinate tomato was observed in V-48 (14650 µM/g FW). Tomato advance lines tested for the fruit total phenolic compound exhibited medium value ranging from 2200 to 9950 µM/g FW, respectively.

Among the indeterminate tomato lines the lowest value for fruit total phenolic compound was observed in a hybrid, NBH-255 (325 µM/g FW). While the highest value was observed in an indeterminate cherry tomato, NI-cherry (14100 µM/g FW). The two semi-determinate tomato lines showed intermediate values for fruit total phenolic compound, AVTO1311 showed a value of 3025 µM/g FW and AVTO1315 showed a value of 3625 µM/g FW, respectively.

## Enzymatic antioxidants

### Ascorbate peroxidase activity

**Figure 4:**
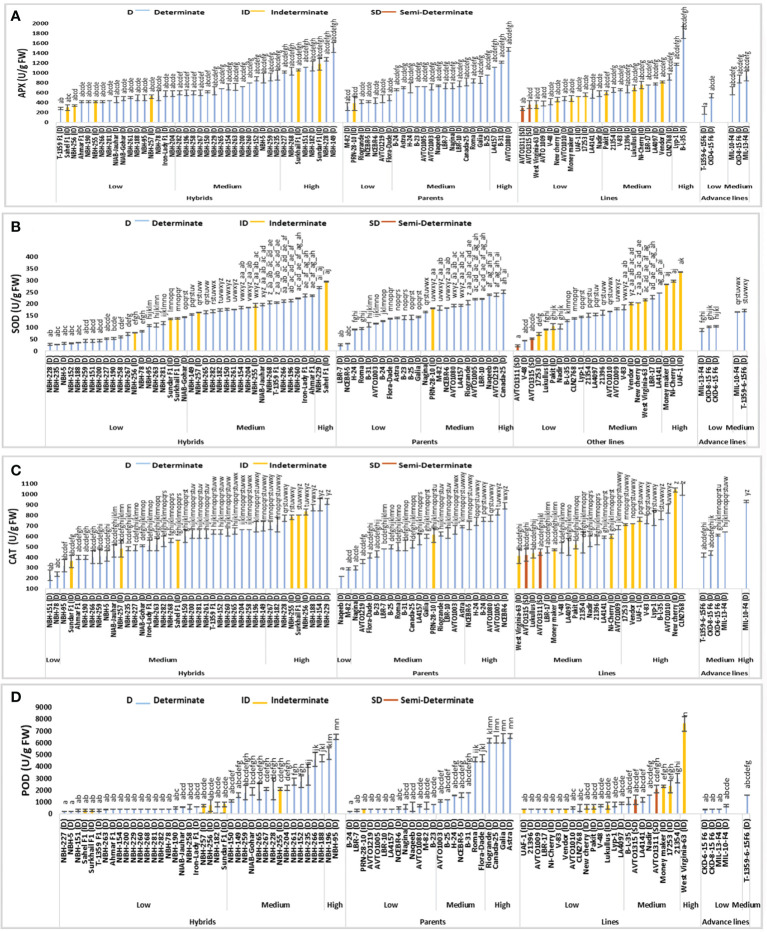
Comparison of fruit **(A)** ascorbate peroxidase (APX), **(B)** superoxide dismutase (SOD), **(C)** catalase (CAT) and **(D)** peroxidase (POD) activities in different tomato genotypes (mean value ± SD). Mean value with varying alphabet differs significantly (p < 0.05, Tukey**’**s HSD).

Significant variation was observed for fruit ascorbate peroxidase (APX) activity among forty tomato hybrids tested. In the low category seventeen tested hybrids were placed with their mean values ranging from 280 to 580 U/g FW (**Figure 4**). The lowest value for determinate tomato was observed in a hybrid, T-1359 F1 (280 U/g FW). In the intermediate category fifteen hybrids were placed ranging from 600 to 960 U/g FW. Eight hybrids were included in the high category with their mean values ranging from 1020 to 1520 U/g FW respectively. The highest value for determinate tomato was observed in hybrid, NBH-149 (1520 U/g FW).

Twenty-four tomato parents were tested for fruit ascorbate per oxidase (APX) activity. Seven parent genotypes showed low values ranging from 320 to 500 U/g FW. The lowest value for determinate tomato was observed in a parent, M-82 (320 U/g FW). In the intermediate category, fourteen tomato parents were placed with their mean values ranging from 660 to 960 U/g FW. Three tomato parent genotypes were placed in high category for fruit ascorbate peroxidase activity, with their mean values ranging from 1120 to 1480 U/g. The highest value for determinate tomato was observed in a parent line, AVTO1080 (1480 U/g FW).

Among twenty-four tested tomato lines, twelve lines were categorized in low category for fruit ascorbate peroxidase (APX) activity. Lowest value for determinate tomato was observed in an inbred line, AVTO1009 (380 U/g FW). The overall range for the lines placed in low category was between 280 to 580 U/g FW. In the intermediate category ten lines were placed with their mean value ranging from 600 to 920 U/g FW. In the high category, only two lines were grouped including a local determinate tomato line, Lyp-1 (1180 U/g FW) and an exotic determinate tomato line, B-L-35 (1860 U/g FW).

Tomato advance lines tested exhibited low and medium values for fruit ascorbate peroxidase (APX) activity. Two advance lines were placed in low category. The lowest value was observed in T-1359-6-15F6, (240 U/g FW), remaining advance lines were placed in medium category ranging from 640 to 940 U/g FW, respectively.

Among sixteen indeterminate tomato lines tested, the lowest value for fruit ascorbate peroxidase (APX) activity was observed in a hybrid Sahel F1 (300 U/g FW) and the highest value was observed in a hybrid Sundar F1 (1180 U/g FW). The two semi-determinate tomato lines showed a low value for fruit ascorbate oxidase activity, with the lowest value observed in a line AVTO1311 (280 U/g FW) followed by a line AVTO1315 (360 U/g FW), respectively.

### Superoxide dismutase activity

Among forty tomato hybrids, twenty showed low values for fruit superoxide dismutase (SOD) activity ranging from 28.02 to 143.34 U/g FW (**Figure 4**). The lowest SOD activity for determinate tomato was observed in a hybrid, NBH-228 (28.02 U/g FW). Eighteen hybrid tomatoes showed intermediate value for fruit SOD activity, ranging from 155.73 to 235.11 U/g FW. For determinate tomatoes highest SOD activity was observed in the hybrid, NBH-229 (269.90 U/g FW).

SOD activity for twelve tomato parents exhibited low values ranging from 25.26 to 144.04 U/g FW. The lowest SOD activity for determinate tomato was observed in a parent line, LBR-7 (25.26 U/g FW). Eleven tomato parents were grouped in the intermediate category for SOD activity with their mean value ranging from 165.27 to 239.21 U/g FW. Canada-25, a determinate tomato parent genotype showed the highest value (251.30 U/g FW) for SOD activity.

Among twenty-four tested tomato lines, ten showed the lowest fruit SOD activity ranging from 21.52 to 144.79 U/g FW. The lowest SOD activity for determinate tomato was observed in a line V-48 (43.21 U/g FW). Eleven lines were grouped in the medium category for SOD activity ranging from 151.06 to 245.50 U/g FW. Three lines were grouped into high category.

Low and medium values were observed for SOD activity among five tested advance lines. Total three exhibited low values ranging from 88.88 to 104.92 U/g FW. While the lowest SOD activity was detected in an advance line, MIL-13 -F4 (88.88 U/g FW). In the medium category two advance lines were grouped.

For sixteen indeterminate tomato genotypes the lowest SOD activity was observed in a line, 17253 (72.28 U/g FW) and the highest was observed in a local line UAF-1 (335.54 U/g FW). The two semi-determinate inbred lines exhibited low SOD activities. An inbred line AVTO1311 showed a value of 21.52 U/g FW. While the semi-determinate inbred line AVTO1315 exhibited an SOD activity of 52.67 U/g FW, respectively.

### Catalase activity

Significant variation was observed for fruit catalase (CAT) activity in the forty tested tomato hybrids (**Figure 4**). In low category two hybrids NBH-151 (230 U/g FW) and NBH-78 (240 U/g FW) were placed. In the medium category twenty-nine hybrids were grouped ranging from 320 to 695 U/g FW. Nine genotypes were grouped into high category for CAT activity ranging from 700 to 930 U/g FW. The highest value for determinate tomato was observed in a local hybrid, NBH-229 (930 U/g FW).

Out of twenty-four tomato parent lines tested for fruit CAT activity, two parents were categorized in low category. The lowest value for determinate tomato was observed in a parent genotype, Naqeeb (220 U/g FW). In the intermediate category sixteen parent their mean values ranging from 300 to 690 U/g FW. Six parents were grouped in the high category, ranging from 700 to 890 (U/g FW). The highest value for determinate tomato was observed in a parent, NCEBR-6 (890 U/g FW).

Tomato lines tested for fruit CAT activity showed medium and high mean values. Out of twenty-four tomato lines tested, fifteen were grouped into medium category ranging from 410 to 680 U/g FW. In the high category for fruit CAT activity nine lines were grouped ranging from 710 to1045 U/g FW and the highest value was observed in CLN2768 (1045 U/g FW).

Among five tomato advance lines tested for fruit CAT activity, four were grouped into medium category ranging from 420 to 640 U/g FW. An advance line MIL-10-F4 was categorized in the high category with the mean value of 930 U/g FW.

Among the indeterminate tomato genotypes, lowest value for fruit CAT activity was observed in a local hybrid Sundar F1 (360 U/g). While the highest mean value was exhibited by a local indeterminate cherry tomato NewCherry (1040 U/g FW). The two semi-determinate lines tested for CAT activity exhibited medium values, whereas AVTO1315 showed a mean value of 420 U/g FW, and AVTO1311 showed mean value of 450 U/g FW, respectively.

### Peroxidase activity

Significant variation was observed among forty tested tomato hybrids for fruit peroxidase (POD) activity (**Figure 4**). In the low category twenty-four genotypes were grouped ranging from 199.80 to 799.20 U/g FW. The lowest value for determinate tomato was observed in a hybrid, NBH-227 (199.80 U/g FW). In the intermediate category fourteen hybrids were placed ranging from 1098.90 to 4695.30 U/g FW. Two tested determinate hybrids NBH-196 (5094.94 U/g FW) and NBH-95 (6496.50 U/g FW) were placed in the high category for fruit POD activity.

The parent genotypes tested for their fruit POD activity exhibited significant differences. In the low category thirteen out of twenty-four genotypes were grouped ranging from 199.80 to 799.20 (U/g FW). Whereas the lowest value for determinate tomato was observed in a parent line, B-24 (199.80 U/g FW). Seven parent genotypes were placed in an intermediate category ranging from 1098.00 to 4695.30 U/g FW. In the high category four parents were grouped ranging from 6193.80 to 6593.40 U/g FW. The highest value for determinate tomato was observed in a parent line, Astra (6593.40 U/g FW).

Out of twenty-four tomato lines tested for fruit POD activity fifteen were categorized into low category ranging from 399.60 to 899.10 (U/g FW). The lowest value for determinate tomato was observed in a line 21396 (399.60 U/g FW). Eight lines were grouped into medium category ranging from 1098.90 to 2997.00 U/g FW. Only one line West Virginia-63 (7592.4 U/g FW) was included in high category.

Total five tomato advance lines tested for POD activity showed low and medium category for POD activity, four of them were grouped into low category ranging from 399.10 to 699.30 (U/g FW). The lowest value was observed in an advance line CKD-6-15 F6 (399.1 U/g FW). In the intermediate category only one advance line T-1359-6-15F6 (1598.40 U/g FW) was placed.

Among the indeterminate tomato lines lowest value for POD activity was observed in a hybrid Sahel F1 (299.70 U/g FW). The highest value for POD activity was observed in an indeterminate line West Virginia-63 (7592.40 U/g FW). The two tested semi-determinate tomato lines AVTO1315 (1198.80 U/g FW) and AVTO1311 (2097.90 U/g FW) were placed in the medium category.

## Hydrolytic enzymes

### Alpha-amylase activity

**Figure 5:**
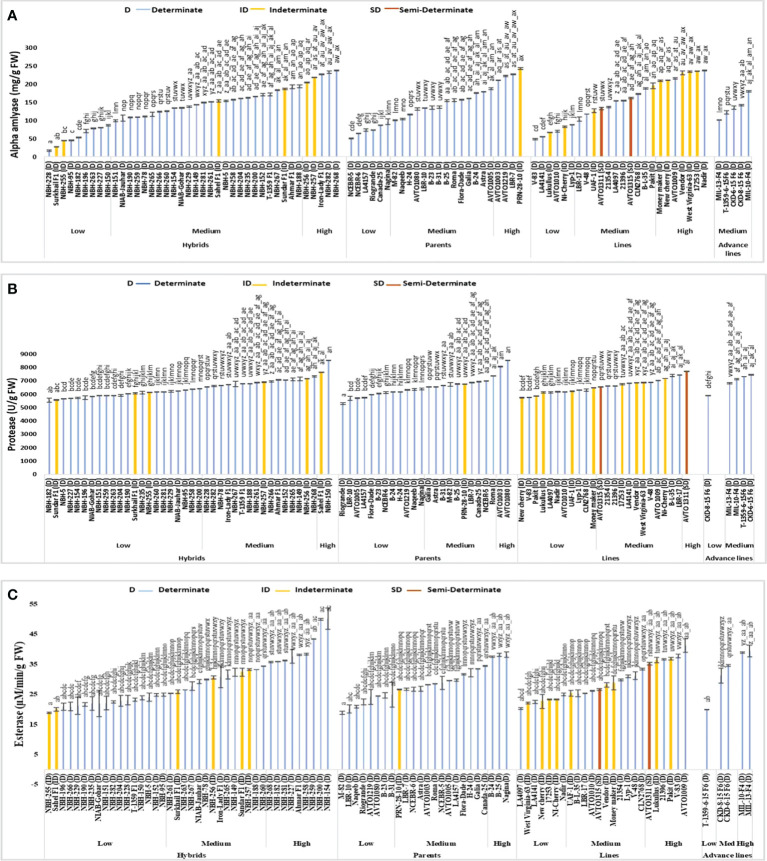
Comparison of fruit **(A)** alpha-amylase, **(B)** protease, and **(C)** esterase activities in different tomato genotypes (mean value ± SD). Mean value with varying alphabet differs significantly (p < 0.05, Tukey**’**s HSD).

Total forty tomato hybrids evaluated for their fruit alpha-amylase activity exhibited significant variations (**Figure 5**). In the low category nine tested hybrids were placed with their mean values ranging from 16.79 to 86.60 mg/g FW, respectively. The lowest value for determinate tomatoes was observed in a local hybrid, NBH-228 (16.79 mg/g FW). In the intermediate category twenty-six hybrids were categorized with their mean values ranging from 100.00 to 194.52 mg/g FW. Five tested hybrids were categorized in high category with their mean values ranging from 204.90 to 238.11 mg/g FW, respectively. The highest value for determinate tomato was observed in a local hybrid, NBH-268 (238.11 mg/g FW).

Among twenty-four tomato parents tested for alpha-amylase activity six showed low mean values ranging from 50.56 to 96.98 mg/g FW. Lowest value for determinate tomato was observed in a parent line, NCEBR-5 (50.56 mg/g FW). In the intermediate category fourteen parents were placed with the mean values ranging from 101.32 to 187.35 mg/g FW. Four parent tomatoes were placed in high category with the mean values ranging from 210.75 to 243.01 (mg/g FW). Highest value for determinate tomato was observed in a parent, PRN-28-10 (243.01 mg/g FW).

The tomato lines evaluated for fruit alpha-amylase activity exhibited significant variation. In the low category six lines were grouped with their mean values ranging from 48.86 to 88.86 mg/g FW, respectively. The lowest value for determinate tomato was observed in a line V-83 (48.86 mg/g FW). In the intermediate category twelve lines were placed with the mean values ranging from 103.58 to 210.00 (mg/g FW). Six tomato lines were grouped into high category with the mean value ranging from 210.56 to 237.73 mg/g FW. The highest value for determinate tomato was observed in a line Nadir (237.73 mg/g FW).

Five tomato advance lines tested for fruit alpha-amylase activity showed medium value ranging from 102.07 to 180.37 mg/g FW. Among the indeterminate tomato lines lowest value was observed in a hybrid Surkhail F1 (27.73 mg/g FW) whereas, the highest value was observed in a parent genotype PRN-28-10 (243.01 mg/g FW). The two semi-determinate line AVTO1311 (133.20 mg/g FW) and AVTO1315 (161.69 mg/g FW) were grouped in the medium category for fruit alpha-amylase activity.

### Protease activity

Hybrids under study exhibited significant variation in the mean value for fruit protease activity (**Figure 5**). In the low category out of a total forty hybrids twenty-two were grouped ranging from 5560 to 6415 U/g FW. The lowest value for determinate tomato was observed in a hybrid, NBH-182 (5560 U/g FW). In the intermediate category sixteen hybrids were grouped ranging from 6535 to 7335 (U/g FW). Two hybrids, Sahel F1(7660 U/g FW) and NBH-150 (8525 U/g FW) were grouped in the high category.

In the low category for fruit protease activity, twelve out of a total twenty-four parents were grouped with their mean value ranging from 5285 to 6400 (U/g FW). The lowest value was observed in a parent Riogrande (5285 U/g FW). In the medium category ten parent genotypes were grouped ranging from 6540 to 7385 (U/g FW). Two parent genotypes were grouped in high category for fruit protease activity including an AVRDC inbred line, AVTO1003 (8070 U/g FW) followed by a line, AVTO1080 (8525 U/g FW).

Among twenty-four tomato lines evaluated for protease activity, eleven showed low values ranging from 5745 to 6490 U/g FW. The lowest value for determinate tomato was observed in a line V-83 (5760 U/g FW). In the intermediate category twelve lines were grouped with their mean values ranging from 6550 to 7430 (U/g FW). The highest value of 7730 U/g FW for protease activity was observed in a line AVTO-1311.

Five tomato advance lines tested for fruit protease activity showed low and medium mean values. The lowest value was observed in a line CKD-8-15 F6 (5910 U/g FW). In the intermediate category four advance lines were grouped with their mean values ranging from 6830 to 7455 (U/g FW).

Among the sixteen indeterminate tomato lines the lowest value was observed in a hybrid Sundar F1 (5575 U/g FW). While the highest value was observed in an indeterminate hybrid Sahel F1 (7660 U/g FW). The semi-determinate line AVTO1315 showed a medium mean value (6550 U/g FW) for protease activity, while the semi-determinate line AVTO1311 (7730 U/g FW) was placed in high category for fruit protease activity.

### Esterase activity

Among forty tomato hybrids tested for fruit esterase activity, seventeen showed low values ranging from 18.85 to 24.87 µM/min/g FW, respectively (**Figure 5**). The lowest value for determinate tomato was observed in a local hybrid, NBH-196 (20.87 µM/min/g FW). In the medium category fourteen hybrids were placed, with their values varying between 25.41 to 34.40 µM/min/g FW. In the high category nine hybrids were grouped with their mean values ranging from 35.70 to 50.15 µM/min/g FW. The highest value for determinate tomato was observed in a hybrid, NBH-154 (50.15 µM/min/g FW).

Tomato parents tested for fruit esterase activity exhibited significant variation. Eight genotypes were grouped into low category with their mean value ranging from 18.85 to 24.64 µM/min/g FW. The lowest value was observed in a parent M-82 (18.85 µM/min/g FW). In the intermediate category thirteen parents were grouped ranging from 26.58 to 34.44 µM/min/g FW. Three genotypes were categorized in high category ranging from 37.50 to 38.13 µM/min/g FW with the highest value observed in Nagina (38.13 µM/min/g FW).

Tomato lines evaluated for fruit esterase activity exhibited significant variation. Out of twenty-four lines total seven were grouped in the low category with the mean value ranging from 20.24 to 24.91 µM/min/g FW. The lowest value was observed in an exotic line LA4097 (20.24 µM/min/g FW). In the intermediate category eleven lines were grouped with their mean values ranging from 25.32 to 33.32 µM/min/g FW. In high category six tomato lines were placed with their mean value ranging from 35.25 to 41.19 µM/min/g FW. The highest value was observed in an AVRDC developed inbred line AVTO1009 (41.19 µM/min/g FW).

Tomato advance lines tested for fruit esterase activity exhibited significant variation. In the low category an advance line T-1359-6-15 F6 (19.92 µM/min/g FW) was placed. Two advance lines CKD-8-15 F6 (31.21 µM/min/g FW) and CKD-6-15 F6 (34.62 µM/min/g FW) showed medium values, while the remaining two were grouped in the high category with the highest value observed in MIL-13-F4 (39.43 µM/min/g FW).

Among sixteen indeterminate lines tested for fruit esterase activity, the lowest value was observed in an indeterminate hybrid, NBH-255 (18.85 µM/min/g FW), while the highest value was observed in a genotype, Pakit (36.83 µM/min/g FW). The two semi-determinate lines showed medium and high values for fruit esterase activity i.e., AVTO1315 showed a value of 26.58 µM/min/g FW, while AVTO1311 exhibited a value of 35.25 µM/min/g FW, respectively.

## Other biochemical assays

### Total soluble sugars

**Figure 6:**
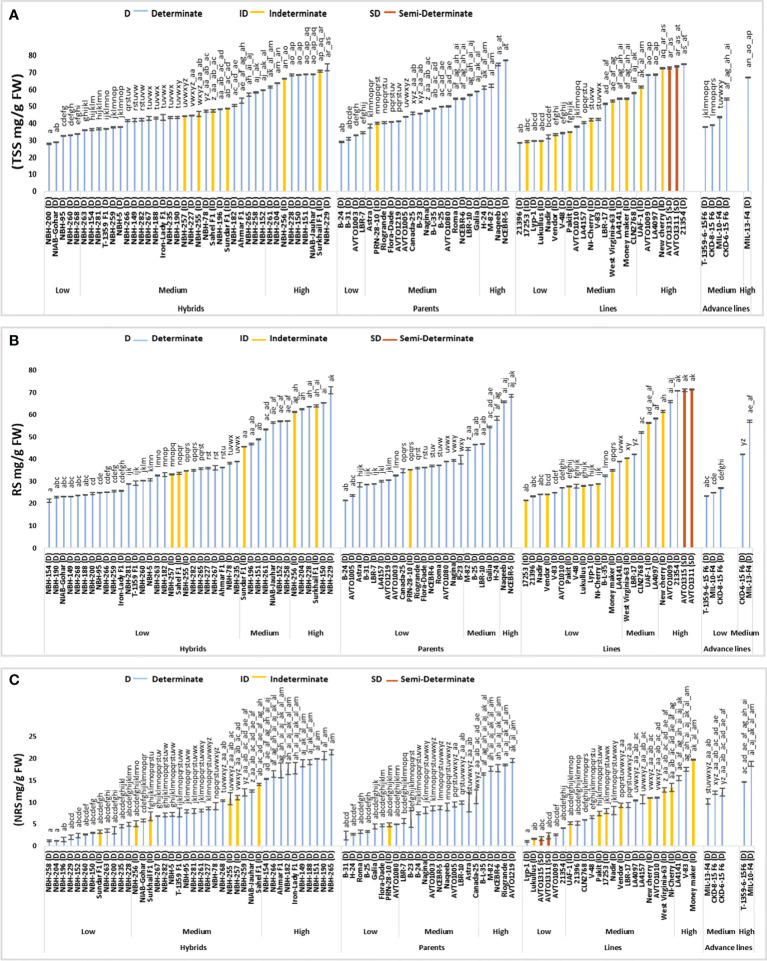
Comparison of fruit **(A)** total soluble sugars (TSS), **(B)** reducing sugars (RS) and **(C)** non-reducing sugars (NRS) in different tomato genotypes (mean value ± SD). Mean value with varying alphabet differs significantly (p < 0.05, Tukey**’**s HSD).

Tomato hybrids tested for fruit total soluble sugar (TSS) showed significant variation (**Figure 6**). In the low category five genotypes were grouped ranging from 28.01 to 33.89 mg/g FW. The lowest value for determinate tomato was observed in a local hybrid, NBH-200 (28.10 mg/g FW). In the intermediate category twenty-six hybrids were grouped ranging from 36.20 to 59.40 mg/g FW. Total nine hybrids were included in high category with their mean values ranging from 61.55 to 73.00 mg/g FW. The highest value for determinate tomato was observed in a hybrid, NBH-229 (73 mg/g FW).

Among twenty-four tomato parents tested for fruit total soluble sugar, four showed low values ranging from 29.00 to 34.55 mg/g FW. The lowest value was observed in a parent line, B-24 (29.00 mg/g FW). In the medium category sixteen parents were grouped ranging from 38.45 to 58.85 (mg/g FW). Four parents were grouped in high category ranging from 61.10 to 77.15 mg/g FW, with the highest value observed in a parent line, NCEBR-5 (77.15 mg/g FW).

Tomato lines tested for fruit total soluble sugars showed significant variation. Seven lines were grouped in the low category with their mean values ranging from 28.51 to 34.40 mg/g FW. The lowest value was observed in a line 21396 (28.51 mg/g FW). In the medium category ten lines were grouped with their mean values ranging from 35.05 to 57.90 mg/g FW. Seven lines were grouped in high category ranging from 61.45 to 74.90 mg/g FW, whereas the highest value was observed in a line 21354 (74.90 mg/g FW).

Tomato advance lines exhibited medium and high value for fruit total soluble sugar. In the medium category four advance lines were grouped ranging from 37.95 to 67.15 mg/g FW. Highest value was observed in an advance line MIL-13-F4 (67.15 mg/g FW).

Among the indeterminate tomato genotypes lowest value was observed in a line, 17253 (29.35 mg/g FW), whereas the highest value for total soluble sugars was observed in a hybrid Surkhail F1 (70.75 mg/g FW). The two semi-determinate lines showed a high value for fruit total soluble sugar AVTO1315 showed a value of 72.65 mg/g FW followed by AVTO1311 (73.60 mg/g FW), respectively.

### Reducing sugar

Among forty tomato hybrids tested for fruit reducing sugars (RS) twenty-seven showed low values ranging from 21.30 to 38.97 mg/g FW (**Figure 6**). The lowest value for determinate tomato was observed in a hybrid, NBH-154 (21.30 mg/g FW). In the intermediate category seven hybrids were grouped with their mean values ranging from 45.57 to 57.15 mg/g FW. Six hybrids were grouped in high category ranging from 61.22 to 70.98 mg/g FW. The highest value for determinate tomato was observed in a hybrid, NBH-229 (70.98 mg/g FW).

Twenty-four tomato parents tested for fruit reducing sugars showed significant variation. In the low category seventeen parents were grouped with the mean values ranging from 21.54 to 39.84 mg/g FW. The lowest value for determinate tomato was observed in a parent line, B-24 (21.54 mg/g FW). In the medium category five parents were grouped with the mean value ranging from 44.58 to 58.41 mg/g FW. Two determinate parents, Naqeeb (65.81 mg/g FW) and NCEBR-5 (68.45 mg/g FW) were grouped in the high category for fruit reducing sugars.

Tomato lines evaluated for their fruit reducing sugars exhibited significant variations. Fourteen lines were grouped in low category with their mean values ranging from 21.42 to 39.05 mg/g FW. The lowest value for the determinate line was observed in 21396 (23.24 mg/g FW). In the intermediate category five lines were grouped ranging from 40.39 to 58.22 mg/g FW. Five tomato lines were categorized in high category with their mean values ranging from 61.50 to 71.26 mg/g FW. The highest value was observed in a determinate line21354 (70.79 mg/g FW).

Out of five advance lines tested for fruit reducing sugars, total three were categorized in low category ranging from 23.35 to 26.95 mg/g FW. While the lowest value was observed in T-1359-6-15 F6 (23.35 mg/g FW). Two advance lines CKD-6-15 F6 (42.09 mg/g FW) and MIL-13-F4 (56.99 mg/g FW) were grouped into the medium category.

The lowest value of fruit reducing sugar for the indeterminate tomato was observed in a line, 17253 (21.42 mg/g FW). While the highest value was observed in a hybrid, Surkhail F1 (63.99 mg/g FW). The two semi-determinate lines AVTO1315 and AVTO 1311 showed high values of 71.00 mg/g FW and 71.26 mg/g FW, respectively.

### Non-reducing sugars

Forty tomato hybrids tested for fruit non reducing sugars (NRS) exhibited significant variation (**Figure 6**). In the low category twelve hybrids were grouped ranging from 1.19 to 4.93 mg/g FW. The lowest value for determinate tomato was observed in a local hybrid, NBH-258 (1.19 mg/g FW). In the intermediate category, eighteen hybrids were placed ranging from 5.07 to 14.03 mg/g FW. Whereas in the high category ten hybrids were grouped, ranging from 15.24 to 21.38 mg/g FW. The highest value of fruit non reducing sugars for determinate hybrids was observed in NBH-265 (21.38 mg/g FW).

The tomato parents tested for their fruit non reducing sugars exhibited significant variation. Out of twenty-four parents tested eight were included in low category ranging from 2.34 to 4.94 mg/g FW. The lowest value for determinate tomato was observed in a parent genotype, B-31 (2.34 mg/g FW). Eleven parents were grouped in the medium category ranging from 5.69 to 11.24 mg/g FW. In the high category five parent lines were grouped ranging from 16.40 to 19.47 mg/g FW. The highest value for determinate tomato was observed in a parent line, AVTO1219 (19.47 mg/g FW).

The tomato lines tested for the fruit non reducing sugars showed significant variation. In the low category six out of a total twenty- four lines were grouped ranging from 1.10 to 4.10 mg/g FW. The lowest value for determinate tomato was observed in a local line Lyp-1 (1.10 mg/g FW). In the medium category fifteen lines were grouped ranging from 5.24 to 13.30 mg/g FW. Three lines showed high values for fruit non reducing sugars ranging from 15.44 to 19.65 (mg/g FW). The highest value for the determinate line was observed in V-83 (17.48 mg/g FW).

Tomato advance lines showed medium and high values for fruit non reducing sugar. In the medium category three advance lines were grouped ranging from 10.15 to 12.25 mg/g FW. Two advance lines were grouped in the high category with the highest value observed in MIL-10-F4 (18.77 mg/g FW).

Sixteen indeterminate tomato lines were tested for fruit non reducing sugar, the lowest value was observed in line Lukullus (1.62 mg/g FW). Whereas, the highest value was observed in an indeterminate variety, Moneymaker (19.65 mg/g FW). The two semi-determinate lines, AVTO1315 (1.65 mg/g FW) and AVTO1311 (2.33 mg/g FW) showed low values for fruit non reducing sugars.

### Malondialdehyde content

**Figure 7:**
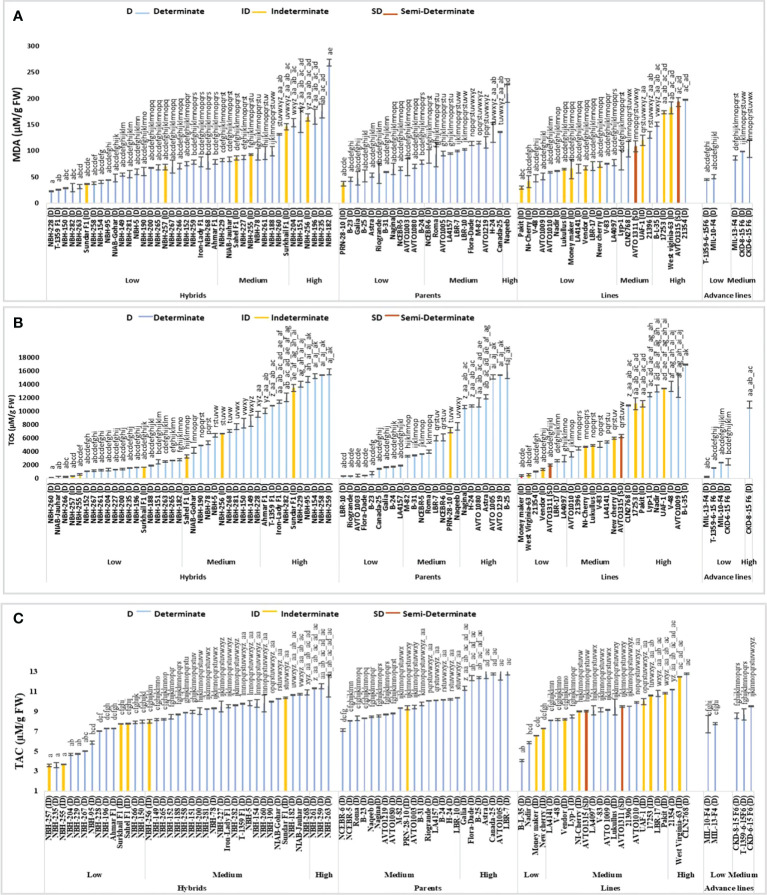
Comparison of fruit **(A)** malondialdehyde (MDA) content, **(B)** total oxidant status (TOS), and **(C)** total antioxidant capacity (TAC) in different tomato genotypes (mean value ± SD). Mean value with varying alphabet differs significantly (p < 0.05, Tukey**’**s HSD).

Forty hybrid tomatoes tested for malondialdehyde (MDA) content exhibited significant variation (**Figure 7**). In the low category twenty-four hybrids were grouped ranging from 22.45 to 78.96 µM/g FW, with the lowest value for determinate tomato observed in NBH-228 (22.45 µM/g FW). In the intermediate category ten hybrids were placed ranging from 82.06 to 145.54 µM/g FW. Total Six hybrids were grouped in the high category for fruit MDA content ranging from 153.29 to 267.87 µM/g FW. The highest value for determinate tomato was observed in a hybrid, NBH-182 (267.87 µM/g FW).

Tomato parents tested for MDA content exhibited significant variation. Out of twenty-four tested tomato parents twelve were grouped in low category ranging from 37.16 to 78.19 µM/g FW. The lowest value for determinate tomato was observed in a parent, B-23 (45.67 µM/g FW). In the medium category eleven parents were grouped, ranging from 89.03 to 135.48 µM/g FW. Only a single determinate tomato parent Naqeeb (209.03 µM/g FW) showed a high value for MDA content.

Tomato lines showed significant variation for fruit MDA content. In the low category fourteen lines were grouped ranging from 29.41 to 77.41 µM/g FW. The lowest value for determinate tomato was observed in a line V-48 (47.22 µM/g FW). In the medium category five lines were grouped ranging from 82.06 to 130.06 µM/g FW. Five lines were placed in the high category for fruit MDA content ranging from 150.96 to 197.41 µM/g FW, respectively. The highest value for determinate tomato was observed in a line 21354 (197.41µM/g FW).

Tomato advance lines showed low and high values for fruit MDA content. In the low category two advance lines were grouped with the lowest value observed in an advance line T-1359-6-15 F6 (44.90 µM/g FW). Whereas, in medium category three advance lines were grouped ranging from 85.93 to 103.74 µM/g FW, respectively.

Among sixteen indeterminate tomato lines tested for fruit MDA content, the lowest value was observed in Pakit (29.41 µM/g FW). Whereas the highest value was observed in an indeterminate line, West Virginia-63 (181.93 µM/g FW). The two semi-determinate line tested showed medium and high values for tomato fruit MDA content, AVTO1311 showed a value of 108.38 µM/g FW, whereas AVTO1315 exhibited a value of 192.77 µM/g FW, respectively.

### Total oxidant status

Out of forty tomato hybrids tested for fruit total oxidant status (TOS) nineteen were classified into a low category with the mean values ranging from 100 to 2775 µM/g FW respectively (**Figure 7**). The lowest value for determinate tomato was observed in a local hybrid, NBH-260 (100 µM/g FW). In the intermediate category eleven hybrids were grouped with their mean values ranging from 3250 to 9525 µM/g FW. Ten tested hybrids were categorized in high category with their mean values ranging from 10050 to 15875 µM/g FW. The highest value of TOS for determinate tomato was observed in a local hybrid, NBH-259 (15875 µM/g FW).

Twenty-four tomato parents involved in the study showed significant variation for fruit TOS. In the low category for fruit TOS nine parents were grouped with their mean values ranging from 325 to 1875 µM/g FW. The lowest value for determinate tomato was observed in the parent line, LBR-10 (325 µM/g FW). In the medium category, eight parents were grouped with their mean values ranging from 3300 to 7750 µM/g FW. In the high category, seven parents were grouped with their mean values ranging from 10600 to 15925 µM/g FW. The highest value for fruit TOS among determinate tomatoes was observed in a parent line, B-25 (15925 µM/g FW).

Twenty-four tomato lines tested for fruit TOS, seven were categorized in the low category with the mean values ranging from 375 to 2950 µM/g FW. Among the determinate tomato lines lowest value for TOS was observed in a line 21354 (1000 µM/g FW). In the medium category for fruit TOS eight tomato lines were grouped with the mean value ranging from 3600 to 6300 µM/g FW. In the high category for tomato fruit TOS nine tomato lines were grouped with their mean values ranging from 10875 to 16950 µM/g FW. The highest value for determinate tomato was observed in a line B-L-35 (16950 µM/g FW).

Tomato advance lines showed low and high values for fruit TOS. In the low category four tomato advance lines were placed ranging from 225 to 2475 µM/g FW. The lowest value was observed in MIL-13-F4 (225 µM/g FW). The highest value of 10975 µM/g FW was observed in the advance line CKD-8-15 -F6, respectively.

Among the indeterminate tomatoes, the lowest value of TOS was observed in a local hybrid, NBH-257 (325 µM/g FW). While the highest value for indeterminate tomato was observed in a local hybrid Sundar F1 (13450 µM/g FW). The semi-determinate lines showed low and medium values for fruit TOS. Semi-determinate line AVTO1311 showed a low value of 1975 µM/g FW, whereas AVTO1315 showed a low value of 6300 µM/g FW, respectively.

### Total antioxidant capacity

Forty tomato hybrids evaluated for their fruit total antioxidant capacity (TAC) values showed significant variation. Fourteen hybrids showed low TAC values ranging from 3.57 to 7.98 µM/g FW (**Figure 7**). The lowest value for determinate tomato was observed in NBH-235 (3.62µM/g FW). In the medium category twenty-three hybrids were grouped ranging from 8.03 to 10.94 µM/g FW. Total three hybrids were grouped into high category ranging from 11.35 to 11.59 µM/g FW. The highest mean value for determinate tomato was observed in a local determinate hybrid, NBH-263 (11.59 µM/g FW).

Tomato parent genotypes showed medium and high values for fruit TAC. In the medium category seventeen out a of total twenty-four parents were grouped with their mean values ranging from 7.14 to 10.37 µM/g FW. The remaining seven tomato parents were placed in the high category ranging from 11.35 to 12.90 µM/g FW. The highest mean value of TAC for determinate tomato was observed in a parent tomato line, LBR-7 (12.90 µM/g FW).

Tomato lines showed significant variation for fruit TAC values. In the low category four out of twenty-four lines were grouped with their mean values ranging from 4.06 to 7.31 µM/g FW. The lowest value for determinate tomato was observed in an exotic line B-L-35 (4.06 µM/g FW). Seventeen lines were grouped in medium category ranging from 8.12 to10.84 µM/g FW. In the high category three lines were grouped with their fruit TAC values ranging from 11.21 to 12.80 µM/g FW. The highest value for determinate tomato was observed in an exotic line CLN2768 (12.80 µM/g FW) respectively.

Tomato advance lines tested for fruit TAC exhibited low and medium values. In the low category, two advance lines were grouped with the lowest value observed in MIL-10-F4 (7.74 µM/g FW). Three advance lines were grouped in medium category ranging from 8.57 to 9.54 µM/g FW.

Among indeterminate tomatoes genotypes tested for fruit TAC, the lowest value was observed in NBH-257 (3.57 µM/g FW), whereas the highest TAC value was observed in an exotic indeterminate line, West Virginia-63 (12.48 µM/g FW). The semi-determinate tomato lines tested for their fruit TAC showed medium values i.e., AVTO1315 showed a value of 9.06 µM/g FW, whereas AVTO1311 showed a value of 9.50 µM/g FW.

## Principal component analysis

**Figure 8:**
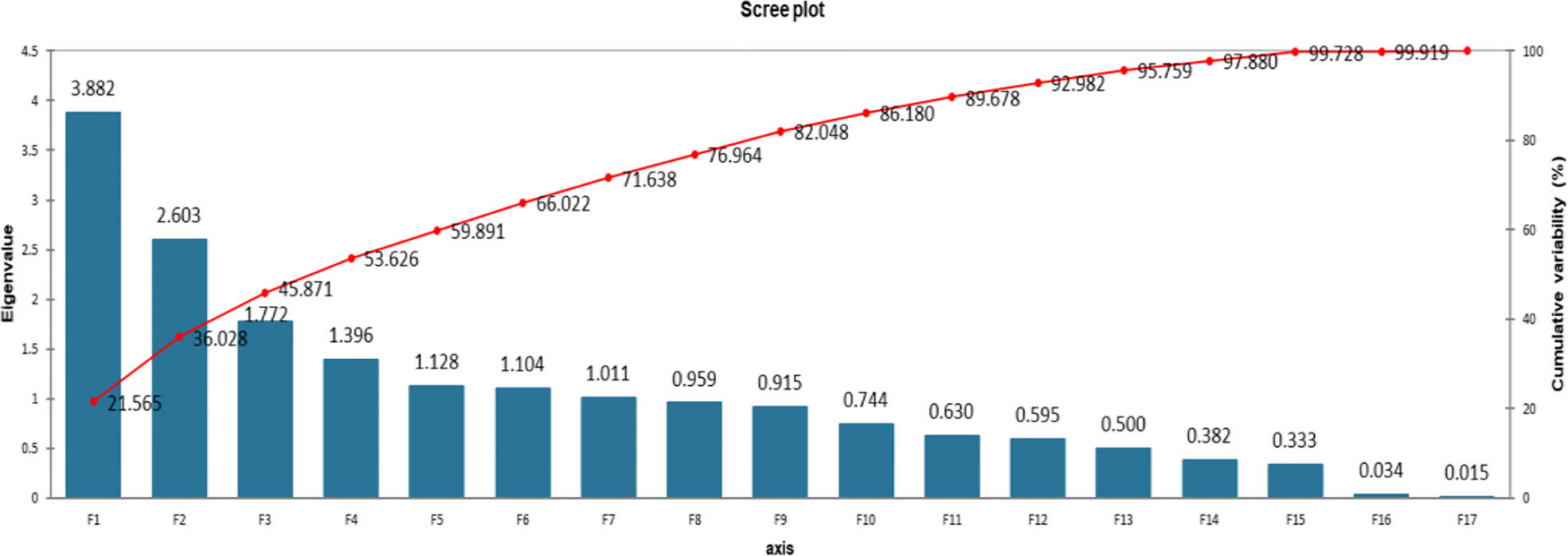
Scree plot **(A)** representing eigenvalue and cumulative variability for parameter under investigation.

Principal component analysis was performed to minimize the dimensionality of datasets in an interpretable manner while preserving all the possible variability among the tested genotypes for studied parameters. The eigenvalue determines an important principal component for further investigation. The principal component with eigen value more than 1 represents about 10% of the total variation (Brejda et al., 2000). Data was subjected to principal component analysis (PCA). Eigen value >1 was the best indicator of the system traits in principal components (Kumar et al., 2019). Scree plot (**Figure 8**) exhibited that, out of total 21 principal components eight (PC-I, PC-II, PC-III, PC-IV, PC-V, PC-VI, and PC-VII) had Eigenvalues > 1 and carried 69.81% of the total cumulative variability. PC-I and PC-II together with cumulative variability of 31.13%, were the largest contributors to the total cumulative variability in the genetic resource. PC-I, PC-II, PC-III, PC-IV, PC-V contributed 50% to the total cumulative variability and PC-I was the major component which explained maximum variation (18.56%) (**Table S1**).

**Figure 9:**
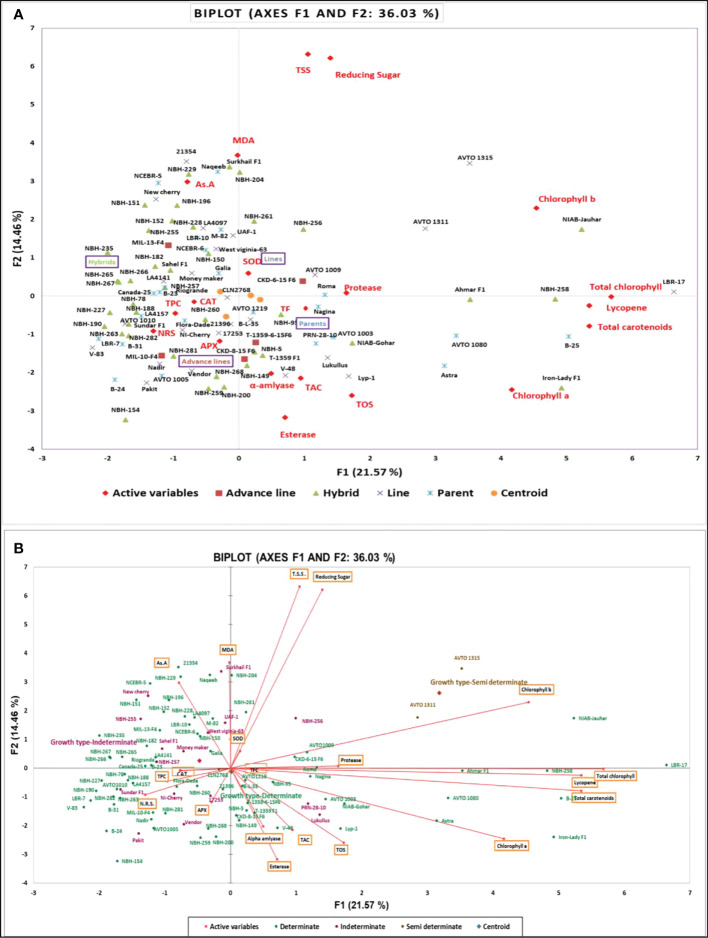
Biplot of the tomato genotypes for first two principal components **(A)** representing centroids of categories hybrid, parents, lines and advance line, and **(B)** representing centroids for growth habit determinate, indeterminate and semi-determinate.

By plotting the PC-I scores (x-axis) against PC-II scores (y- axis), a genotype by trait (G-T) biplot was generated for all the genotypes and their traits (**Figure 9**). A multiple traits visual comparison among the genotypes and the interrelationship between the traits was effectively revealed in the genotype by trait (G-T) biplot. Important information was extracted from the angles between the vectors and the distance of the variables from the origin of the biplot. The angle of the vector with principal component axis determined its contribution to that PC. The more parallel is a vector to the principal component axis the more it contributed to that specific PC. In the correlation circle, vector length represented the representativeness quality in the investigated PCA dimensions. Correlation between the two traits was considered positive, if the angle between these traits was less than 90, whereas correlation was considered negative if the angle between the two vector traits was greater than 90. The right angle between the traits represented that the traits were independent of each other *(* Shah et al., 2020 *)*. Considering the angle between the vectors and principal component axis a positive correlation was observed between total chlorophyll, lycopene, total carotenoids, chlorophyll b and chlorophyll a. Moreover, total chlorophyll, total carotenoids, lycopene, chlorophyll b and chlorophyll a exhibited positive factor loading 0.477, 0.450, 0.450, 0.380, 0.351 with positive contribution to PC-I (**Table S1**). Whereas TSS, RS, MDA, ascorbic acid, chlorophyll b and POD with factor loading 0.512, 0.504, 0.311, 0.253, 0.208 and 0.153 showed positive correlation and had greater contribution to PC-II. NRS, SOD, POD and chlorophyll b with factor loading 0.415, 0.412, 0.241 and 0.207 showed positive contribution to PC-III. Among the individual category of tomato genotypes (hybrids, parents, lines and advance lines), the parents and the lines had factor scores 0.325 and 0.186 in PC-I, whereas the hybrids had factor score 0.114 in PC-II. Moreover, categories of tomato genotypes based on the growth habit revealed that semi-determinate and determinate tomato genotypes had the factor scores 3.184 and 0.015, whereas semi-determinate and indeterminate tomato with factor scores 0.252 and 2.612 exhibited greater influence on the traits having significant contribution in PC-II respectively.

## Correlation analysis

Correlation (Pearson test) for all investigated biochemical traits was performed with 95% confidence interval. Lycopene showed a significant positive correlation with chlorophyll a, chlorophyll b, total carotenoids and total chlorophyll (**Table S3**). Whereas chlorophyll a showed a significant positive correlation with Total antioxidant capacity (TAC), esterase, total oxidant status (TOS), total chlorophyll and total carotenoids and significant negative correlation with non-reducing sugars. Chlorophyll b showed a significant positive correlation with total chlorophyll, total carotenoids, reducing sugar and total soluble sugars. Whereas the total carotenoids exhibited significant positive correlation with total chlorophyll and TOS. A significant positive correlation was observed between total chlorophyll, protease, and TOS. Total soluble sugars (TSS), ascorbic acid (ASA) and malondialdehyde (MDA) showed a significant positive correlation with reducing sugars and significant negative correlation with non-reducing sugars. TSS exhibited significant positive correlation with MDA. Moreover, non-reducing sugars represented significant positive correlation with the superoxide dismutase (SOD). Alpha-amylase showed a significant positive correlation with protease. Whereas AsA showed significant positive correlation with MDA and significant negative correlation with protease. MDA showed a significant negative correlation with TOS. Esterase exhibited a significant positive correlation with TAC and significant negative correlation with SOD respectively.

## Discussion

Tomato (*Solanum lycopersicum* L.) is a rich source of nutrients, antioxidants and bioactive compounds (Salehi et al., 2019; Ali et al., 2021). These nutrients helps accomplish numerous body functions such as lipid profile maintenance, blood circulation stimulation and detoxification of bone structure (Campestrini et al., 2019; Vats et al., 2020). A direct relationship was found between tomato fruit intake and its anticancer activity (Wargovich, 2000). The presence of high concentrations of natural antioxidant in tomato fruit plays significant role in inhibiting reactive oxygen species (ROS) by free radicals scavenging, prevents cellular proliferation and apoptosis, plays role in enzymatic activities modulation and signal transduction pathways (Agarwal and Rao, 2000; Hossen et al., 2017; Navarro-González et al., 2018). The present study aims to evaluate the comprehensive nutritional and antioxidant potential of the tomato fruit. Different biochemical assays were performed to find nutritionally rich genotypes that can be further manipulated in crop improvement programs.

Tomato offers a key dietary source of carotenoids including lycopene and β-carotene (Manoharan et al., 2017). Lycopene is considered a strong lipophilic antioxidant in tomatoes. It is the most important free radical scavenger of all carotenoids (Shi and Maguer, 2000). It has been reported to enhance glutathione levels and the overall activities of antioxidant enzymes. The antioxidant activity of lycopene can protect lipids, DNA and other macromolecules from damage (Anlar and Bacanli, 2020). Lycopene accounts for 80 to 95% of the total carotenoid content in tomatoes (Karniel et al., 2020). In the present study, comparatively 13% of the forty tomato hybrids and 6% of the other fifty-three genotypes showed high fruit lycopene content. Previously reported lycopene content in fresh tomato fruit ranged between 1.86–14.62 mg/100g which was in accordance with the present findings (Frusciante et al., 2007). In general, the highest lycopene content was found in a local developed determinate hybrid NIAB-Gohar. The increased lycopene content in the hybrid NIAB-Gohar compared with the parents (LBR-7 and Nagina) may be attributed to heterosis. Whereas heterosis is a commonly used natural biological phenomena in which heterozygotes with different genetic bases are produced between two or more parents by hybridization. Hybrids are superior to parents related to growth rate, yield, quality, viability and disease resistance (Hochholdinger and Hoecker, 2007). Heterosis is commonly utilized in plants including vegetables to enhance yield, stress tolerance, quality and nutritional properties (Wang et al., 2021; Yu et al., 2021; Zhang et al., 2021).

Carotenoids are a chief dietary reservoir of vitamin A, which is obtained from bioconversion of β-carotene retinol into pro-vitamin A (Tang, 2010). For total carotenoid content 13% of the forty hybrids and 9% of the remaining fifty-three genotypes exhibited high values. Generally, the maximum total carotenoid was observed in the hybrid local NIAB-Gohar exhibiting heterosis compared with the parents (Nagina and LBR-7). In a previous report the total carotenoid content in fresh tomato fruit varied between 3.87-18 mg/100 g which was in accordance the present study (Frusciante et al., 2007; Alda et al., 2009; Pal et al., 2018; Górecka et al., 2020). Notably present finding validates that the local hybrid NIAB-Gohar could be a potential source for improving tomato fruit lycopene and total carotenoid contents.

Carotenoids as a precursor of aroma compounds indirectly affect flavor, whereas chlorophylls play role in the production of sugar through photosynthesis. Carotenoid accumulation during ripening determines chlorophyll degradation together with the fruit color (Aono et al., 2021). Both these traits have been and will continue to be of great importance in plant breeding efforts (Manoharan et al., 2017). In the present investigation 8% of the hybrids and 6% of the remaining genotypes exhibited high total chlorophyll content. Among hybrids, NIAB-Jauhar showed the highest total chlorophyll content (194.74 µg/100g FW) which was higher than the better parents, (LBR-10, 84.71 µg/100g FW and Roma, 116.28 µg/100g FW) indicating hybrid vigor. The activities of domestication and improvement in the cultivated tomato has jeopardized flavor and nutritional quality parameters in tomato fruit. Recent emphasis on breeding strategies aims on flavor associated chemicals like acids, sugar and aroma compounds (Aono et al., 2021).

Ascorbic acid (AsA) is an important non-enzymatic antioxidant. It functions as an antioxidant by scavenging reactive oxygen species (Jameel et al., 2021). The high concentration of AsA content in tomatoes plays a fundamental role in different aspects related to plant life and human health (Di Matteo et al., 2010). Moreover, AsA also acts as plant growth modulator *via* hormone signaling (Khalid and Hameed, 2017). The human body is unable to make AsA by its own because its biosynthesis is prevented at the final stage. Consequently, food crops containing a high level of AsA content are essential for human health (Davuluri et al., 2005; Hancock and Viola, 2005). In the present investigation AsA content was found higher in 15% of the forty tomato hybrids and 13% of the remaining fifty-three genotypes. The highest AsA content was found in a line 21396 (435.25 µg/g FW), which was in accordance with the previously reported AsA content in fresh tomato fruit i.e., 122 to 475 µg/g FW (Frusciante et al., 2007; Di Matteo et al., 2010; Pal et al., 2018). Whereas, among hybrids the highest AsA was observed in a local determinate hybrid NBH-152 (400.75 µg/g FW), which was higher than both the parents AVTO1005 and Naqeeb (343.75 and 371.25 µg/g FW) showing heterosis. Currently, in different crops hybrids are cultivated globally owing to their superior performances and adaptability to various environments compared with inbred. Heterosis can be exploited to improve fruit nutritional composition in tomato (Fortuny et al., 2021).

Flavonoids are the major naturally occurring phenols (Evans, 2009). In the present investigation, generally 15% of the forty hybrids and 13% of the other fifty-three genotypes showed high total flavonoid content (TFC). Notably in the case of two local indeterminate cherry tomato UAF-1 (4009.19 µg/100 g FW) and NI-Cherry (2487.94 µg/100 g FW) TFC was found maximum. In the previous study, TFC in fresh tomato fruit was reported to vary between 1150 to 8160 µg/100 g FW (Dewanto et al., 2002; Martínez-Valverde et al., 2002; Raffo et al., 2002; Dumas et al., 2003; Raffo et al., 2006; Frusciante et al., 2007) which validates the present finding. Generally, the Total phenolic compounds (TPC) in 3% of the hybrids and 11% of the remaining genotypes was found higher. Notably again the two cherry tomatoes NI-Cherry (14100 µM/g FW) and 17253 (13275 µM/g FW) were included in high category for TPC (George et al., 2004). In a previous study it was found that the TPC in the pulp of three cherry tomato cultivars to be higher than other larger fruit tomato varieties (George et al., 2004). Moreover, another finding explained that the higher amount of TPC in cherry tomatoes compared to cultivars with larger fruits is mainly due to the higher skin to volume ratio of cherry varieties, which could improve their phenolic content especially flavonols, as these compounds occur inside the skin of the fruit (Stewart et al., 2000). In a previous report, TPC in tomato fruit varied between 37 to 86 mg GAE 100/g FW (Delgado-Vargas et al., 2018), whereas in another report the TPC of tomatoes was estimated between 267.18 and 775.04 mg GAE/kg FW (Park et al., 2020). Although various studies have described the phenolic content in tomatoes however comparison of findings is not feasible in most of the cases because of diverse methods of extraction as well as various solvents and evaluating techniques have been used.

Plant develops several ROS-scavenging mechanism to counter adverse impacts of ROS accumlation and to establish appropriate ROS homeostasis. Antioxidant enzymes such as superoxide dismutase (SOD), peroxidase (POD), ascorbate peroxidase (APX) and catalase (CAT) plays a vital role in ROS scavenging and maintain homestasis in fruit and plants (Apel and Hirt, 2004; Vall-Llaura et al., 2022). ROS apart from being considered a toxic by-product, also plays significant role as a signaling molecule in various biological processes including fruit development, ripening and responses to various abiotic and biotic stresses (Fichman and Mittler, 2020). Superoxide dismutase (SOD) is a vital primary enzyme that removes superoxide radicles, transforming them into hydrogen peroxide and dioxygen. In the present study, comparatively 2% of the forty hybrid tomatoes and 8% of the other fifty-three genotypes divulged high SOD activity. Generally the highest activity was observed in a cherry tomatoes UAF-1 (335.54 U/g FW) and among hybrids the highest value was observed in Sahel F1 (294.46 U/g FW) which was in accordance with the previously reported SOD activity in tomato fruit (Gautier et al., 2010). Ascorbate peroxidase (APX) reduces hydrogen peroxide by using ascorbate as the electron donor and regulates the accumulation of toxic level of hydrogen peroxide under stress conditions (Salandanan et al., 2009). In the present investigation, comparatively 23% of the forty tomato hybrids and 9% of the other fifty-three genotypes showed high APX activity. In general, maximum APX activity was detected in a local determinate line, B-L-35 (1860 U/g FW) and among hybrids, NBH-149 showed the maximum activity (1520 U/g FW) which was found to be greater than both the parents NCEBR-5 and AVTO1219 (420 and 480 U/g FW), that could be attributed to heterosis. The APX activity at the red ripe stage of tomato fruit was reported between 200 to 1200 (µmol min-1 g-1 FW) respectively (Gautier et al., 2010). Catalase (CAT) catalyzes hydrogen peroxide dismutation in oxygen and water (Rani et al., 2004; Ortega-Ortiz et al., 2007). In the present research, comparatively 23% of the forty tomato hybrids and 30% of the remaining genotypes showed high CAT activity, whereas maximum activity was observed for a line CLN2768 (1045 U/g FW) and again hybrid vigor was observed in the hybrid NBH-229 (930 U/g FW) for CAT activity compared with the better parents (Naqeeb 220 U/g FW and AVTO1080 770 U/g FW). In a previous report, a medicinal plant *Peganum harmala* used in the treatment of various diseases such as diabetes, depression, cough, and some other human ailment divulged lower catalase activity (555 U/g) (Ahmed et al., 2020) compared with the present finding. Peroxidase (POD) contributes to phenol oxidation, hormone catabolism, lignin polymerization, cell wall, proteins and polysaccharides intercrossing, defense against pathogens and fruit ripening. In fruit ripening, and mainly during climacterium POD is increased along with the cellulase enzymes and polygalacturonase (Robinson and Eskin, 1991; Ortega-Ortiz et al., 2007). In the present study, comparatively 5% of the hybrid tomatoes and 9% of the remaining fifty-three genotypes exhibited high POD activity. In general, the highest POD activity was observed for an indeterminate exotic line West Virginia-63 (7592.40 U/g FW), and a local determinate hybrid NBH-95 (6496.50 U/g FW) respectively. The high value of POD activity for hybrid NBH-95 compared with the parent Naqeeb and M-82 (and 599.40 and 699.30 U/g FW) could be attributed to heterosis.

Different enzymes present in the tomato pulp, plays a significant part in the nutrient turnover in tomato pulp at various maturity stages. Previously it has been reported that hydrolytic and proteolytic enzymes perform some physiological functions during maturation and fruit senescence (Desai and Deshpande, 1978; Hashinaga et al., 1983). Hydrolytic enzymes such as alpha-amylase, esterase and protease in living organisms specifically break up larger molecules into smaller molecule by the process of hydrolysis, a water molecule is added to a substance during the process (Wong et al., 2020). Hydrolytic enzymes also behave as a secondary source of antioxidant by utilizing damaged molecules and repairing DNA (Pradedova et al., 2011). Amylase enzyme hydrolysis starch to obtain monomeric carbohydrates. Starch degradation occurs during cereal seed germination. Hydrolytic enzymes are responsible for the breakdown, it is generally believed that phosphorylases are not involved in this activity, whereas α-amylase performs a significant role during the breakdown of local starch granules (Perata et al., 1992). Alpha-amylase help catalyzes hydrolysis of 4-glycosidic linkages, together with internal a-1 in starch to obtain products such as maltose and glucose. The enzyme can be obtained from plants, animals, and microorganisms (Sundarram and Murthy, 2014). In general, 13% of the tomato hybrids and 21% of the remaining genotypes explicated high alpha-amylase activity. The maximum alpha-amylase activity was observed in an indeterminate parent PRN-28-10 (243.01 mg/g FW), which was relatively higher than the alpha-amylase activity (164.90 mg/g dry wt.) found in a medicinal plant, T. simplex (Ahmed et al., 2020), and fairly lower than the alpha-amylase activity (292.70 mg/g s. wt.) of wheat flour (Khalid and Hameed, 2017). Among hybrid NBH-268 (238.11 mg/g FW) showed maximum alpha-amylase activity, which was higher than better parents (Riogrande, 74.52 mg/g FW and AVTO1003, 210.75 mg/g FW) that can be ascribed to hybrid vigor. To utilize carbon and energy present in the starch polymer it must be degraded to smaller digestible sugars prior to its conversion into individual glucose unit (Alam et al., 2006). In different stages of plant life cycle, proteases play a vital role in the overall procedure of protein turnover (Rani et al., 2012). Proteases being a protein hydrolytic enzymes, act upon proteinaceous substance present in the cell wall delivering amides and amino acids (Alam et al., 2006). In the present study 5% of the tomato hybrids and 6% of the remaining genotypes revealed high protease activity. Generally, the highest activity was observed in a local hybrid NBH-150 and determinate line AVTO1080 (8525 U/g FW) respectively. Hybrid NBH-150 showed protease activity higher than the better parent NCEBR-5 (7005 U/g FW) that could be accredited to hybrid vigor. According to previous studies, during ripening protease activity increases in kiwi and tomato fruit (Hashinaga et al., 1983; Alam et al., 2006). Increased protease activity during maturation has also been detected in passion fruit juice (Hashinaga et al., 1978). High protease activity in the ripening stage may be ascribed to protein catabolism which is connected to leaf senescence (Dilley, 1970). The present investigation validates higher protease activity in tomato fruit at the red ripe stage. Esterases are found in a variety of living organisms and have the capacity to catalyze the synthesis of ester bond and their hydrolysis from different substrates (Zhong et al., 2020). In the present study, 23% of the tomato hybrids and 21% of the remaining genotypes showed maximum esterase activity. In general, the highest activity was observed in a local hybrid NBH-154 (50.15 µM/min/g FW) respectively which was relatively higher than the previously reported esterase activity of 14.30 mg/g in a medicinal plant, *Zygophyllum fabago* (Ahmed et al., 2020).

Sugars accounts for an essential component of tomato fruit, as they regulate sweetness and flavor. High sugars are needed for the best flavor. Tomato fruit contains mainly glucose and fructose, whereas sucrose is present in the trace amount (Tadesse et al., 2012). Fructose and glucose accounts for about 65% of total soluble solid in tomato fruit (Turhan and Şeniz, 2009). Different genotypes under investigation showed significant variation in their sugar content. In general, 25% of the hybrid tomatoes and 23% of the remaining genotypes showed high Total soluble sugars (TSS). The highest TSS content was observed for a determinate parent NCEBR-5 (77.15 mg/g FW) and among hybrids NBH-229 (73.00 mg/g FW) showed highest total TSS. Previously reported TSS content in fresh tomato fruit ranged between 1.7% to 4.7% (17 to 47 mg/g FW) respectively (Melkamu et al., 2008; Turhan and Şeniz, 2009; Tadesse et al., 2012), which was fairly lower than the TSS content in the highest category of the present investigation. The low and medium categories of the present finding were in accordance with the previously reported TSS content of 1.4 to 5% (14 – 50 g/g FW) in tomato fruit (Dai et al., 2016) respectively.

Reducing sugars (RS) in tomato contributes to the sweet taste, whereas total sugars exhibits a significant fraction of fruit dry weight and are influenced by seasons and variety (Tadesse et al., 2012). Present investigation explicated that comparatively 15% of the forty tomato hybrids and 13% of the remaining genotypes showed high RS content, whereas the highest RS content was observed for a semi-determinate AVRDC line AVTO1311 (71.26 mg/g) and among hybrids NBH-299 (70.98 mg/g FW) showed hybrid vigor compared with parents (AVTO1080, 39.05 mg/g FW and Naqeeb, 65.81 mg/g FW). Reducing sugars (RS) content in fresh tomato fruit was reported to be 1.1 to 4.1% (11-41 mg/g) which was relatively lower than the RS content in the highest category (60 to 72 mg/g FW) of the present investigation, while RS content in the low and medium category (20- 59 mg/g FW) of the present study validates the previous report (Ereifej et al., 1997; Tadesse et al., 2012). In general, 25% of the forty tomato hybrids and 19% of the remaining fifty three genotypes showed high values for non reducing sugars (NRS) content. The highest value was observed for a determinate hybrid NBH 265 (21.39 mg/g) showed a value greater than the better parent (AVTO1003, 8.59 mg/g FW and NCEBR-5, 8.69 mg/g FW). The NRS content in low and medium categories (1.1 to 14.4 mg/g FW) of the present study confirmed the previous findings whereas non reducing sugar (NRS) content for tomato fruit was detected to vary between 0.11 to 14 mg/g respectively (Ereifej et al., 1997; Tadesse et al., 2012).

Malondialdehyde (MDA) content is usually used as a lipid peroxidation marker and is mainly utilized as a sign of damage to the plant membranes (Morales and Munné-Bosch, 2019). In general, 15% of the tomato hybrids and 11% of the remaining genotypes showed high MDA content. The highest MDA content was in the local determinate hybrid NBH-182 (267.87 µM/g FW), and this high value could be ascribed to heterosis. MDA can play a significant positive role in the acclimation process rather than damage by activation of regulatory genes associated with the plant defense system (Tounekti et al., 2011).

The total antioxidant status (TAS) is used to estimate the overall antioxidant capacity in an organism (Sevindik, 2018), whereas the total oxidant status (TOS) is used to estimate the overall oxidation state of the living organism (Vaiserman et al., 2020). According to the present investigation 25% of the tomato hybrids and 32% of the remaining genotypes showed high total oxidant status (TOS). In general, the highest TOS was observed in a determinate line B-L-35 (16950 µM/g FW) and determinate hybrid NBH-259 (15875 µM/g FW). The hybrid showed TOS higher than both parents B-23 and AVTO1005 (775 and 15100 µM/g FW). The TOS level of tomato genotypes was higher than the TOS reported in Asteraceae (3020 µM/L) and Lamiaceae (5260 mM/L) families (Caf et al., 2018). According to a previous report there was a progressive enhancement in oxidative stresses throughout fruit development. Moreover, during the early period of fruit ripening, the antioxidant system is efficient to protect tomato fruit from progressive oxidative damages. But at later stages the ROS scavenging system is not effective enough to manage the production system resulting in ROS accumulation (Mondal et al., 2004). The high TOS value for the tomato fruit could be because of its red ripe stage. In the present study, the free radical scavenging activity was divulged in the form of inhibition of free radical ABTS. Comparatively 8% of the hybrid tomatoes and 19% of the other fifty-three genotypes showed maximum TAC. Generally, the maximum TAC was observed in a parent line LBR-7 (12.90 µM/g FW) and among hybrids in NBH-263 (11.59 µM/g FW), which was higher than the parents AVTO1080 and B-24 (8.81 and 10.17 µM/g FW) that could be attributed to heterosis. The TAC of the tomato genotypes in the present investigation was found to be relatively lower than the previously reported tomato fruit TAC (14-27 µmol g−1) (Sahlin et al., 2004). However it was quite higher than the TAC (0.48 -1.18 μmol/g−1 and 0.054 – 0.209 µmol 100 g−1) of tomato fruit reported in another study (Zhou and Yu, 2006; Erge and Karadeniz, 2011).

To simplify the explanation of a larger amount of data and to obtain significant information from the data set, a multivariant statistical method called Principal Component Analysis (PCA) is applied (Rathinavel, 2018). PCA explained the significance of the main contributor to the total variation at the differentiation axis. Eigenvalues helps in interpreting the important factor which can be taken. The numerical closer to unity in PC- I having the largest absolute value influence the grouping considerably more, in contrast to the ones with smaller absolute values that are closer to zero (Bhanupriya et al., 2014; Mishra et al., 2015). In the present study, PCA was launched for all variables to understand the pattern of variation. Out of twenty-one principal components, total eight principal components with eigenvalues >1 elucidated 69.81% of the total variation. PC-I, PCII, PC-III, PC-IV, PC-V, PC-VI, and PC-VII revealed 18.56, 12.58, 8.74, 7.46, 6.54, 5.61, 5.33, and 4.99% variability, respectively (**Table S1**). The traits like total chlorophyll, lycopene, total carotenoids, chlorophyll a, chlorophyll b, α amylase, TAC, protease, esterase, TOS, SOD and TFC showed considerable positive contribution in PC- I with positive factor loadings. On the basis of individual loading, a single variable is normally selected from these recognized groups (Mishra et al., 2015). Total chlorophyll showed the highest factor loading (0.941), followed by total carotenoids (0.889), and lycopene (0.888). Total chlorophyll could be the finest choice with the highest contribution in PC-I, whereas Chlorophyll b, total chlorophyll, RS, TSS, AsA, MDA, protease, SOD, POD exhibited considerable positive contribution in PC- II. So, PC- II was related to diversity among genotypes due to TSS (0.832) and RS (0.819) with their positive influence (**Figure 9**). Distance of the genotype from biplot origin estimated genotypic differences relating to the grand mean, thus distances of the genotypes from the origin can be a good indicator of genotypes with superior and inferior performance in the environment. The genotypes found away from the biplot’s origin can be a good performers (Hagos and Abay, 2013) and the genotypes nearer to a particular trait show best performance related to the corresponding trait. The correlation analysis further confirmed the PCA results by exhibiting strong positive and strong negative association between the traits that are closer and far from each other in the PC axis (**Table S3**). In general parents and lines showed a positive contribution in PC- I and the hybrids showed positive contribution in PC- II with positive factor scores (**Table S2**). Therefore, parents and lines could be a potential source for traits like lycopene, chlorophylls, total carotenoids, alpha-amylase, TAC, protease, esterase, TOS and TFC, whereas hybrids could be a potential source for the traits such as total soluble sugars, reducing sugars, MDA, ascorbic acid, chlorophyll b, POD and SOD, respectively. Moreover, semi-determinate and determinate tomato genotypes showed positive factor score in PC-I, thus the genotypes with semi-determinate and determinate growth habits could be a potential source for the above-mentioned traits with greater influence in PC-I, while the semi-determinate and indeterminate tomato genotypes showed significant contribution to the trait performing superior in PC- II. More specifically the hybrids NIAB-Jauhar, Iron-lady F1, NBH-258, Ahmar F1 and NIAB-Gohar, the parents H-24, B-25, AVTO1080, and Astra as well as the lines LBR-17, AVTO1315, AVTO1311 and Lyp-1 found far away from biplot origin with positive factor score in PC-I. Consequently, these genotypes could be a potential source for the traits with better performance in PC-I (**Table S2**). Whereas the hybrids Surkhail F1, NBH-204, NBH-229, NBH-151, NBH-196, NBH-152, NBH-261, NBH-228, NIAB-Jauhar, NBH-256, NBH-255, the lines 21354, AVTO1315, Newcherry, LA4097, AVTO1311 and UAF-1 together with the parents Naqeeb, NCEBR-5, M-82 and LBR-10 were found far away from biplot origin with positive factor score in PC- II. Hence, the above-mentioned genotypes could be a finest choice for the traits with positive influence in PC- II, respectively. Present results demonstrates that principal component analysis along with genetic resource characterization help indicate traits of interest for scheming breeding strategies.

## Conclusion

The present finding concludes that tomato genotypes including hybrids, parents and lines have prominent antioxidant potential and bioactive compounds. For the investigated traits including pigment, hydrolytic enzymes, TOS, TAC and TFC it was found that determinate and semi-determinate tomatoes, category parents and lines and the following genotypes NIAB-Jauhar, Iron-lady F1, NBH-258, H-24, B-25, AVTO1080, Astra, LBR-17, AVTO1315, AVTO1311 and Lyp-1 showed an excellent potential. Moreover for the traits including sugars, AsA, MDA, POD and SOD the indeterminate and semi-determinate tomatoes, category hybrids and the following genotypes Surkhail F1, NBH-204, NBH-229, NBH-151, NBH-196, 21354, AVTO1315, Newcherry, LA4097, AVTO1311, UAF-1, Naqeeb, NCEBR-5, M-82 and LBR-10 exhibited an outstanding performance. Hybrids exhibited superior performance for the investigated traits compared with the parent. The identified growth types, categories and genotypes with superior activity for investigated traits, can be utilized in breeding programs to establish specific breeding strategies and to improve the desirable traits in tomatoes. Moreover, the identified genotypes with higher antioxidant potential and nutritionally rich bioactive compounds can be utilized as a source to improve human health and prevent various chronic degenerative diseases. Moreover, tomato genotypes can also be consumed directly as a natural source of antioxidants to improve endogenous immune system.

## Data availability statement

The original contributions presented in the study are included in the article/**Supplementary Material**. Further inquiries can be directed to the corresponding author.

## Author contributions

BR conducted the overall experiment, analytical work, data collection, data organization, result compilation, write-up, and revision of the manuscript. AH contributed to designing, and finalization of basic idea related to the experiment, overall supervision during wet lab work, data analysis using statistical software, revisions, and finalization of the manuscript. MS aided in providing the tomato germplasm and technical advice. All authors have significant contributions in improving the submitted article. All authors contributed to the article and approved the submitted version.

## Funding

The research work was conducted by the support provided by Nuclear Institute for Agriculture and Biology, Jhang Road, Faisalabad Pakistan.

## Conflict of interest

The authors declare that the research was conducted in the absence of any commercial or financial relationships that could be construed as a potential conflict of interest.

## Publisher’s note

All claims expressed in this article are solely those of the authors and do not necessarily represent those of their affiliated organizations, or those of the publisher, the editors and the reviewers. Any product that may be evaluated in this article, or claim that may be made by its manufacturer, is not guaranteed or endorsed by the publisher.

## References

[B1] Agarwal S. Rao A. V. (2000). Tomato lycopene and its role in human health and chronic diseases. Cmaj 15, e0231612. doi: 10.1371/journal.pone.0231612 PMC80172 11022591

[B2] Ahmed A. Hameed A. Saeed S. (2020). Biochemical profile and bioactive potential of wild folk medicinal plants of zygophyllaceae from balochistan, Pakistan. bioRxiv. doi: 10.1101/2020.03.30.016212 PMC7444594 32810139

[B3] Ainsworth E. A. Gillespie K. M. (2007). Estimation of total phenolic content and other oxidation substrates in plant tissues using folin–ciocalteu reagent. Nat. Protoc. 2, 875–877. doi: 10.1038/nprot.2007.102 17446889

[B4] Alam M. Rahman M. H. Mamun M. Ahmad I. Islam K. (2006). Enzyme activities in relation to sugar accumulation in tomato. Proceedings Pakistan Acad. Sci. 43, 241.

[B5] Alda L. M. Gogoasa I. Bordean D.-M. Gergen I. Alda S. Moldovan C.. (2009). Lycopene content of tomatoes and tomato products. J. Agroaliment. Processes Technol. 15, 540–542.

[B6] Ali M. Y. Sina A. Khandker S. S. Neesa L. Tanvir E. Kabir A.. (2021). Nutritional composition and bioactive compounds in tomatoes and their impact on human health and disease: A review. Foods 10, 45. doi: 10.3390/foods10010045 PMC7823427 33375293

[B7] Anlar H. G. Bacanli M. (2020). "Lycopene as an antioxidant in human health and diseases,". Pathology, 247–254. doi: 10.1016/B978-0-12-815972-9.00024-X 33032809

[B8] Anza M. Riga P. Garbisu C. (2006). Effects of variety and growth season on the organoleptic and nutritional quality of hydroponically grown tomato. J. Food Qual. 29, 16–37. doi: 10.1111/j.1745-4557.2006.00053.x

[B9] Aono Y. Asikin Y. Wang N. Tieman D. Klee H. Kusano M. (2021). High-throughput chlorophyll and carotenoid profiling reveals positive associations with sugar and apocarotenoid volatile content in fruits of tomato varieties in modern and wild accessions. Metabolites 11, 398. doi: 10.3390/metabo11060398 34207208 PMC8233878

[B10] Apel K. Hirt H. (2004). Reactive oxygen species: metabolism, oxidative stress, and signal transduction. Annu. Rev. Plant Biol. 55, 373–399. doi: 10.1146/annurev.arplant.55.031903.141701 15377225

[B11] Asensio E. Sanvicente I. Mallor C. Menal-Puey S. (2019). Spanish Traditional tomato. effects of genotype, location and agronomic conditions on the nutritional quality and evaluation of consumer preferences. Food Chem. 270, 452–458. doi: 10.1016/j.foodchem.2018.07.131 30174071

[B12] Beecher G. R. (1998). Nutrient content of tomatoes and tomato products. Proc. Soc. Exp. Biol. Med. 218, 98–100. doi: 10.3181/00379727-218-44282a 9605204

[B13] Beers R. F. Sizer I. W. (1952). A spectrophotometric method for measuring the breakdown of hydrogen peroxide by catalase. J. Biol. Chem. 195, 133–140. doi: 10.1016/S0021-9258(19)50881-X 14938361

[B14] Bhanupriya B. Satyanarayana N. Mukherjee S. Sarkar K. (2014). Genetic diversity of wheat genotypes based on principal component analysis in gangetic alluvial soil of West Bengal. J. Crop Weed 10, 104–107.

[B15] Borguini R. G. Ferraz Da Silva Torres E. A. (2009). Tomatoes and tomato products as dietary sources of antioxidants. Food Rev. Int. 25, 313–325. doi: 10.1080/87559120903155859

[B16] Brejda J. J. Moorman T. B. Karlen D. L. Dao T. H. (2000). Identification of regional soil quality factors and indicators i. central and southern high plains. Soil Sci. Soc. America J. 64, 2115–2124. doi: 10.2136/sssaj2000.6462115x

[B17] Caf F. Kiliç Ö. Algül S. (2018). Evaluation of total antioxidant status, total oxidant status and oxidative stress index of some economically important plants from Turkey. Prog. IN Nutr. 20, 145–152. doi: 10.23751/pn.v20i1-S.6125

[B18] Campestrini L. H. Melo P. S. Peres L. E. Calhelha R. C. Ferreira I. C. Alencar S. M. (2019). A new variety of purple tomato as a rich source of bioactive carotenoids and its potential health benefits. Heliyon 5, e02831. doi: 10.1016/j.heliyon.2019.e02831 31763483 PMC6859294

[B19] Chance B. Maehly A. (1957). Methods in enzymol Vol. 4. Eds. Colowick S. P. Kaplan N. O. (New York: Academic Press, Inc.), 273.

[B20] Chen G.-X. Asada K. (1989). Ascorbate peroxidase in tea leaves: occurrence of two isozymes and the differences in their enzymatic and molecular properties. Plant Cell Physiol. 30, 987–998. doi: 10.1093/oxfordjournals.pcp.a077844

[B21] Coyago-Cruz E. Corell M. Moriana A. Mapelli-Brahm P. Hernanz D. Stinco C. M.. (2019). Study of commercial quality parameters, sugars, phenolics, carotenoids and plastids in different tomato varieties. Food Chem. 277, 480–489. doi: 10.1016/j.foodchem.2018.10.139 30502174

[B22] Dai Z. Wu H. Baldazzi V. Van Leeuwen C. Bertin N. Gautier H.. (2016). Inter-species comparative analysis of components of soluble sugar concentration in fleshy fruits. Front. Plant Sci. 7, 649. doi: 10.3389/fpls.2016.00649 27242850 PMC4872523

[B23] Davuluri G. R. Van Tuinen A. Fraser P. D. Manfredonia A. Newman R. Burgess D.. (2005). Fruit-specific RNAi-mediated suppression of DET1 enhances carotenoid and flavonoid content in tomatoes. Nat. Biotechnol. 23, 890–895. doi: 10.1038/nbt1108 15951803 PMC3855302

[B24] Delgado-Vargas F. Sicairos-Medina L. Y. Luna-Mandujan A. G. López-Angulo G. Salazar-Salas N. Y. Vega-García M. O.. (2018). Phenolic profiles, antioxidant and antimutagenic activities of solanum lycopersicum var. cerasiforme accessions from Mexico. CyTA Journal Food 16, 715–722. doi: 10.1080/19476337.2018.1481146

[B25] Desai B. Deshpande P. (1978). Effects of stage of maturity on some physical and biochemical constituents and enzyme activities of banana (Musa paradisiaca linn.) fruits. Mysore J. Agric. Sci 12, 193–201.

[B26] Dewanto V. Wu X. Adom K. K. Liu R. H. (2002). Thermal processing enhances the nutritional value of tomatoes by increasing total antioxidant activity. J. Agric. Food Chem. 50, 3010–3014. doi: 10.1021/jf0115589 11982434

[B27] Dhindsa R. S. Plumb-Dhindsa P. Thorpe T. A. (1981). Leaf senescence: correlated with increased levels of membrane permeability and lipid peroxidation, and decreased levels of superoxide dismutase and catalase. J. Exp. Bot. 32, 93–101. doi: 10.1093/jxb/32.1.93

[B28] Dilley D. (1970). Enzymes.(In) the biochemistry of fruits and their products. Acad. Press London 179, 179–207.

[B29] Di Mascio P. Kaiser S. Sies H. (1989). Lycopene as the most efficient biological carotenoid singlet oxygen quencher. Arch. Biochem. Biophys. 274, 532–538. doi: 10.1016/0003-9861(89)90467-0 2802626

[B30] Di Matteo A. Sacco A. Anacleria M. Pezzotti M. Delledonne M. Ferrarini A.. (2010). The ascorbic acid content of tomato fruits is associated with the expression of genes involved in pectin degradation. BMC Plant Biol. 10, 1–11. doi: 10.1186/1471-2229-10-163 20691085 PMC3095297

[B31] Dixit V. Pandey V. Shyam R. (2001). Differential antioxidative responses to cadmium in roots and leaves of pea (Pisum sativum l. cv. azad). J. Exp. Bot. 52, 1101–1109. doi: 10.1093/jexbot/52.358.1101 11432926

[B32] Dogan H. Ercİşlİ S. Temim E. Hadziabulic A. Tosun M. Yilmaz S.. (2014). Diversity of chemical content and biological activity in flower buds of a wide number of wild grown caper (Capparis ovata desf.) genotypes from Turkey. Comptes Rendus L Academie Bulgare Des. Sci. 67, 1593–1600.

[B33] Drapeau G. R. (1976). “[38] protease from staphyloccus aureus,” in Methods in enzymology (New York:Academic Press), 469–475. 10.1016/s0076-6879(76)45041-3 1012010

[B34] Dubois M. Gilles K. Hamilton J. Rebers P. Smith F. (1951). A colorimetric method for the determination of sugars. Nature 168, 167–167. doi: 10.1038/168167a0 14875032

[B35] Dumas Y. Dadomo M. Di Lucca G. Grolier P. (2003). Effects of environmental factors and agricultural techniques on antioxidantcontent of tomatoes. J. Sci. Food Agric. 83, 369–382. doi: 10.1002/jsfa.1370

[B36] Elbadrawy E. Sello A. (2016). Evaluation of nutritional value and antioxidant activity of tomato peel extracts. Arabian J. Chem. 9, S1010–S1018. doi: 10.1016/j.arabjc.2011.11.011

[B37] Ereifej K. Shibli R. Ajlouni M. Hussain A. (1997). Physico-chemical characteristics and processing quality of newly introduced seven tomato cultivars into Jordan in comparison with local variety. J. Food Sci. Technol. 34, 171–174.

[B38] Erel O. (2004). A novel automated direct measurement method for total antioxidant capacity using a new generation, more stable ABTS radical cation. Clin. Biochem. 37, 277–285. doi: 10.1016/j.clinbiochem.2003.11.015 15003729

[B39] Erel O. (2005). A new automated colorimetric method for measuring total oxidant status. Clin. Biochem. 38, 1103–1111. doi: 10.1016/j.clinbiochem.2005.08.008 16214125

[B40] Erge H. S. Karadeniz F. (2011). Bioactive compounds and antioxidant activity of tomato cultivars. Int. J. Food Properties 14, 968–977. doi: 10.1080/10942910903506210

[B41] Evans W. (2009). Treaseosy. 16th Ed (Edinburgh, London, New York, Philadelphia, St Louis, Sydney, Toronto: Saunders/Elsevier).

[B42] Fichman Y. Mittler R. (2020). Rapid systemic signaling during abiotic and biotic stresses: is the ROS wave master of all trades? Plant J. 102, 887–896. doi: 10.1111/tpj.14685 31943489

[B43] Fortuny A. P. Bueno R. A. Pereira Da Costa J. H. Zanor M. I. Rodríguez G. R. (2021). Tomato fruit quality traits and metabolite content are affected by reciprocal crosses and heterosis. J. Exp. Bot. 72, 5407–5425. doi: 10.1093/jxb/erab222 34013312

[B44] Frusciante L. Carli P. Ercolano M. R. Pernice R. Di Matteo A. Fogliano V.. (2007). Antioxidant nutritional quality of tomato. Mol. Nutr. Food Res. 51, 609–617. doi: 10.1002/mnfr.200600158 17427261

[B45] García-Hernández J. Hernández-Pérez M. Peinado I. Andrés A. Heredia A. (2018). Tomato-antioxidants enhance viability of l. reuteri under gastrointestinal conditions while the probiotic negatively affects bioaccessibility of lycopene and phenols. J. Funct. Foods 43, 1–7. doi: 10.1016/j.jff.2017.12.052

[B46] Gautier H. Lopez-Lauri F. Massot C. Murshed R. Marty I. Grasselly D.. (2010). Impact of ripening and salinity on tomato fruit ascorbate content and enzymatic activities related to ascorbate recycling. Funct. Plant Sci. Biotechnol. 4, 66–75.

[B47] Gaweł S. Wardas M. Niedworok E. Wardas P. (2004). Malondialdehyde (MDA) as a lipid peroxidation marker. Wiadomosci lekarskie (Warsaw Poland: 1960) 57, 453–455. 15765761

[B48] George B. Kaur C. Khurdiya D. Kapoor H. (2004). Antioxidants in tomato (Lycopersium esculentum) as a function of genotype. Food Chem. 84, 45–51. doi: 10.1016/S0308-8146(03)00165-1

[B49] Giannopolitis C. N. Ries S. K. (1977). Superoxide dismutases: I. occurrence in higher plants. Plant Physiol. 59, 309–314. doi: 10.1104/pp.59.2.309 16659839 PMC542387

[B50] Górecka D. Wawrzyniak A. Jędrusek-Golińska A. Dziedzic K. Hamułka J. Kowalczewski P. Ł.. (2020). Lycopene in tomatoes and tomato products. Open Chem. 18, 752–756. doi: 10.1515/chem-2020-0050

[B51] Grygorieva O. Klymenko S. Kuklina A. Vinogradova Y. Vergun O. Sedlackova V. H.. (2021). Evaluation of lonicera caerulea l. genotypes based on morphological characteristics offruits germplasm collection. Turkish J. Agric. Forestry 45, 850–860. doi: 10.3906/tar-2002-14

[B52] Hagos H. G. Abay F. (2013). AMMI and GGE biplot analysis of bread wheat genotypes in the northern part of Ethiopia. J. Plant Breed. Genet. 1, 12–18.

[B53] Hameed A. Iqbal N. Malik S. A. Syed H. Ahsanul-Haq M. (2005). Age and organ specific accumulation of ascorbate in wheat (Triticum aestivum l.) seedlings grown under etiolation alone and in combination with oxidative stress. Caderno de Pesquisa série Biologia 17, 51–63

[B54] Hancock R. D. Viola R. (2005). Improving the nutritional value of crops through enhancement of l-ascorbic acid (vitamin c) content: rationale and biotechnological opportunities. J. Agric. Food Chem. 53, 5248–5257. doi: 10.1021/jf0503863 15969504

[B55] Harma M. Harma M. Erel O. (2005). Oxidative stress in women with preeclampsia. Am. J. Obstet. Gynecol. 192, 656–657. doi: 10.1016/j.ajog.2004.07.094 15696019

[B56] Hashinaga F. Sawa D. Itoo S. (1978). Protease in the juice of passion fruit (Passiflora edulis sims). J. Japanese Soc. Hortic. Sci. 47, 282–288. doi: 10.2503/jjshs.47.282

[B57] Hashinaga F. Yamato F. Itoo S. (1983). Partial purification and characterization of protease from passion fruit juice Mem. Fac. Agric. Kagoshima Univ.

[B58] Heath R. L. Packer L. (1968). Photoperoxidation in isolated chloroplasts: I. kinetics and stoichiometry of fatty acid peroxidation. Arch. Biochem. Biophys. 125, 189–198. doi: 10.1016/0003-9861(68)90654-1 5655425

[B59] Hochholdinger F. Hoecker N. (2007). Towards the molecular basis of heterosis. Trends Plant Sci. 12, 427–432. doi: 10.1016/j.tplants.2007.08.005 17720610

[B60] Hossen M. S. Ali M. Y. Jahurul M. Abdel-Daim M. M. Gan S. H. Khalil M. I. (2017). Beneficial roles of honey polyphenols against some human degenerative diseases: A review. Pharmacol. Rep. 69, 1194–1205. doi: 10.1016/j.pharep.2017.07.002 29128800

[B61] Huang D. Ou B. Prior R. L. (2005). The chemistry behind antioxidant capacity assays. J. Agric. Food Chem. 53, 1841–1856. doi: 10.1021/jf030723c 15769103

[B62] Ighodaro O. Akinloye O. (2018). First line defence antioxidants-superoxide dismutase (SOD), catalase (CAT) and glutathione peroxidase (GPX): Their fundamental role in the entire antioxidant defence grid. Alexandria J. Med. 54, 287–293. doi: 10.1016/j.ajme.2017.09.001

[B63] Jaleel C. A. Riadh K. Gopi R. Manivannan P. Ines J. Al-Juburi H. J.. (2009). Antioxidant defense responses: physiological plasticity in higher plants under abiotic constraints. Acta Physiologiae Plantarum 31, 427–436. doi: 10.1007/s11738-009-0275-6

[B64] Jameel S. Hameed A. Shah T. M. (2021). Biochemical profiling for antioxidant and therapeutic potential of Pakistani chickpea (Cicer arietinum l.) genetic resource. Front. Plant Sci. 12, 574. doi: 10.3389/fpls.2021.663623 PMC8076736 33927742

[B65] Karniel U. Koch A. Zamir D. Hirschberg J. (2020). Development of zeaxanthin-rich tomato fruit through genetic manipulations of carotenoid biosynthesis. Plant Biotechnol. J. 18, 2292–2303. doi: 10.1111/pbi.13387 32320515 PMC7589248

[B66] Khalid A. Hameed A. (2017). Seed biochemical analysis based profiling of diverse wheat genetic resource from Pakistan. Front. Plant Sci. 8, 1276. doi: 10.3389/fpls.2017.01276 28775731 PMC5517496

[B67] Kumar A. Kumar A. Ranjan R. Kumar S. Rajani K. Singh P. (2019). Principal component analysis of agro-morpho-genetic traits in desi chickpea (Cicer arietinum l.). SP, 362–365.

[B68] Lee S.-H. Ahsan N. Lee K.-W. Kim D.-H. Lee D.-G. Kwak S.-S. (2007). Simultaneous overexpression of both CuZn superoxide dismutase and ascorbate peroxidase in transgenic tall fescue plants confers increased tolerance to a wide range of abiotic stresses. J Plant Physiol 164, 1626–1638. 17360071 10.1016/j.jplph.2007.01.003

[B69] Lichtenthaler H. K. Wellburn A. R. (1983). Determinations of total carotenoids and chlorophylls a and b of leaf extracts in different solvents (United kingdom:Portland Press).

[B70] Lin J.-Y. Tang C.-Y. (2007). Determination of total phenolic and flavonoid contents in selected fruits and vegetables, as well as their stimulatory effects on mouse splenocyte proliferation. Food Chem. 101, 140–147. doi: 10.1016/j.foodchem.2006.01.014

[B71] Manoharan R. K. Jung H.-J. Hwang I. Jeong N. Kho K. H. Chung M.-Y.. (2017). Molecular breeding of a novel orange-brown tomato fruit with enhanced beta-carotene and chlorophyll accumulation. Hereditas 154, 1–8. doi: 10.1186/s41065-016-0023-z 28096780 PMC5226094

[B72] Martínez-Valverde I. Periago M. J. Provan G. Chesson A. (2002). Phenolic compounds, lycopene and antioxidant activity in commercial varieties of tomato (Lycopersicum esculentum). J. Sci. Food Agric. 82, 323–330. doi: 10.1002/jsfa.1035

[B73] Melkamu M. Seyoum T. Woldetsadik K. (2008). Effects of pre-and post harvest treatments on changes in sugar content of tomato. Afr. J. Biotechnol. 7, 1139–1144.

[B74] Mishra C. Tiwari V. Satish-Kumar V. G. Kumar A. Sharma I. (2015). Genetic diversity and genotype by trait analysis for agromorphological and physiological traits of wheat (Triticum aestivum l.). Sabrao J. Breed. Genet. 47, 40–48.

[B75] Mondal K. Sharma N. Malhotra S. Dhawan K. Singh R. (2004). Antioxidant systems in ripening tomato fruits. Biol. Plantarum 48, 49–53. doi: 10.1023/B:BIOP.0000024274.43874.5b

[B76] Morales M. Munné-Bosch S. (2019). Malondialdehyde: facts and artifacts. Plant Physiol. 180, 1246–1250. doi: 10.1104/pp.19.00405 31253746 PMC6752910

[B77] Navarro-González I. García-Alonso J. Periago M. J. (2018). Bioactive compounds of tomato: Cancer chemopreventive effects and influence on the transcriptome in hepatocytes. J. Funct. Foods 42, 271–280. doi: 10.1016/j.jff.2018.01.003

[B78] Nguyen M. L. Schwartz S. J. (1999). Lycopene: Chemical chemical and biological properties: Developing nutraceuticals for the new millenium. Food Technol. (Chicago) 53, 38–45.

[B79] Noor R. Mittal S. Iqbal J. (2002). Superoxide dismutase–applications and relevance to human diseases. Med. Sci. Monit.: Int. Med. J. Exp. Clin. Res. 8, RA210–RA215. 12218958

[B80] Ortega-Ortiz H. Benavides-Mendoza A. Mendoza-Villarreal R. Ramírez-Rodríguez H. De Alba Romenus K. (2007). Enzymatic activity in tomato fruits as a response to chemical elicitors. J. Mexican Chem. Soc. 51, 141–144.

[B81] Pal R. Hedau N. Kant L. Pattanayak A. (2018). Functional quality and antioxidant properties of tomato genotypes for breeding better quality varieties. Electronic J. of Plant Breed. doi: 10.5958/0975-928X.2018.00001.7

[B82] Park H. Kim Y.-J. Shin Y. (2020). Estimation of daily intake of lycopene, antioxidant contents and activities from tomatoes, watermelons, and their processed products in Korea. Appl. Biol. Chem. 63, 1–11. doi: 10.1186/s13765-020-00534-w

[B83] Perata P. Pozueta-Romero J. Akazawa T. Yamaguchi J. (1992). Effect of anoxia on starch breakdown in rice and wheat seeds. Planta 188, 611–618. doi: 10.1007/BF00197056 24178396

[B84] Pradedova E. Isheeva O. Salyaev R. (2011). Classification of the antioxidant defense system as the ground for reasonable organization of experimental studies of the oxidative stress in plants. Russian J. Plant Physiol. 58, 210–217. doi: 10.1134/S1021443711020166

[B85] Proteggente A. R. Pannala A. S. Paganga G. Buren L. V. Wagner E. Wiseman S.. (2002). The antioxidant activity of regularly consumed fruit and vegetables reflects their phenolic and vitamin c composition. Free Radical Res. 36, 217–233. doi: 10.1080/10715760290006484 11999391

[B86] Raffo A. La Malfa G. Fogliano V. Maiani G. Quaglia G. (2006). Seasonal variations in antioxidant components of cherry tomatoes (Lycopersicon esculentum cv. Naomi F1). J. Food Composition Anal. 19, 11–19. doi: 10.1016/j.jfca.2005.02.003

[B87] Raffo A. Leonardi C. Fogliano V. Ambrosino P. Salucci M. Gennaro L.. (2002). Nutritional value of cherry tomatoes (Lycopersicon esculentum cv. Naomi F1) harvested at different ripening stages. J. Agric. Food Chem. 50, 6550–6556. doi: 10.1021/jf020315t 12381148

[B88] Rani K. Rana R. Datt S. (2012). Review on latest overview of proteases. Int. J. Curr. Life Sci. 2, 12–18.

[B89] Rani P. Unni K. M. Karthikeyan J. (2004). Evaluation of antioxidant properties of berries. Indian J. Clin. Biochem. 19, 103–110. doi: 10.1007/BF02894266 23105465 PMC3454186

[B90] Rathinavel K. (2018). Principal component analysis with quantitative traits in extant cotton varieties (Gossypium hirsutum l.) and parental lines for diversity. Curr. Agric. Res. J. 6, 54. doi: 10.12944/CARJ.6.1.07

[B91] Robinson D. S. Eskin N. (1991). Oxidative enzymes in foods. sole distributor in the USA and Canada (London: Elsevier Applied Science).

[B92] Sahlin E. Savage G. Lister C. (2004). Investigation of the antioxidant properties of tomatoes after processing. J. Food Composition Anal. 17, 635–647. doi: 10.1016/j.jfca.2003.10.003

[B93] Salandanan K. Bunning M. Stonaker F. Külen O. Kendall P. Stushnoff C. (2009). Comparative analysis of antioxidant properties and fruit quality attributes of organically and conventionally grown melons (Cucumis melo l.). HortScience 44, 1825–1832. doi: 10.21273/HORTSCI.44.7.1825

[B94] Salehi B. Sharifi-Rad R. Sharopov F. Namiesnik J. Roointan A. Kamle M.. (2019). Beneficial effects and potential risks of tomato consumption for human health: An overview. Nutrition 62, 201–208. doi: 10.1016/j.nut.2019.01.012 30925445

[B95] Saran P. L. Singh S. Solanki V. Choudhary R. Manivel P. (2021). Evaluation of asparagus adscendens accessions for root yield and shatavarin IV content in India. Turkish J. Agric. Forestry 45, 475–483. doi: 10.3906/tar-2006-42

[B96] Sevindik M. (2018). Investigation of oxidant and antioxidant status of edible mushroom *Clavariadelphus truncatus* . Mantar Dergisi 9, 165–168.

[B97] Shah T. M. Imran M. Atta B. M. Ashraf M. Y. Hameed A. Waqar I.. (2020). Selection and screening of drought tolerant high yielding chickpea genotypes based on physio-biochemical indices and multi-environmental yield trials. BMC Plant Biol. 20, 1–16. doi: 10.1186/s12870-020-02381-9 32303179 PMC7164285

[B98] Shi J. Maguer M. L. (2000). Lycopene in tomatoes: chemical and physical properties affected by food processing. Crit. Rev. Food Sci. Nutr. 40, 1–42. doi: 10.1080/10408690091189275 10674200

[B99] Sies H. (1991). Oxidative stress: Oxidants and antioxidants (New York and London: Academic Press).

[B100] Stewart A. J. Bozonnet S. Mullen W. Jenkins G. I. Lean M. E. Crozier A. (2000). Occurrence of flavonols in tomatoes and tomato-based products. J. Agric. Food Chem. 48, 2663–2669. doi: 10.1021/jf000070p 10898604

[B101] Sundarram A. Murthy T. P. K. (2014). α-amylase production and applications: a review. J. Appl. Environ. Microbiol. 2, 166–175. doi: 10.12691/jaem-2-4-10

[B102] Tadesse T. Workneh T. S. Woldetsadik K. (2012). Effect of varieties on changes in sugar content and marketability of tomato stored under ambient conditions. Afr. J. Agric. Res. 7, 2024–2030. doi: 10.5897/AJAR11.1216

[B103] Tang G. (2010). Bioconversion of dietary provitamin a carotenoids to vitamin a in humans. Am. J. Clin. Nutr. 91, 1468S–1473S. doi: 10.3945/ajcn.2010.28674G 20200262 PMC2854912

[B104] Tounekti T. Vadel A. Oñate M. Khmeira H. BOSCH S. (2011). Salt induced oxidative stress in rosemary plants: damage or protection. Environ. Exp. Bot. 71 298–305. doi: 10.1016/j.envexpbot.2010.12.016

[B105] Turhan A. Şeniz V. (2009). Estimation of certain chemical constituents of fruits of selected tomato genotypes grown in Turkey. Afr. J. Agric. Res. 4, 1086–1092.

[B106] Uçan U. Uğur A. (2021). Acceleration of growth in tomato seedlings grown with growth retardant. Turkish J. Agric. Forestry 45, 669–679. doi: 10.3906/tar-2011-4

[B107] Ulewicz-Magulska B. Wesolowski M. (2019). Total phenolic contents and antioxidant potential of herbs used for medical and culinary purposes. Plant Foods Hum. Nutr. 74, 61–67. doi: 10.1007/s11130-018-0699-5 30374852 PMC6422988

[B108] Vaiserman A. Koliada A. Zayachkivska A. Lushchak O. (2020). Nanodelivery of natural antioxidants: An anti-aging perspective. Front. Bioeng. Biotechnol. 447. doi: 10.3389/fbioe.2019.00447 PMC6965023 31998711

[B109] Vall-Llaura N. Fernández-Cancelo P. Nativitas-Lima I. Echeverria G. Teixidó N. Larrigaudière C.. (2022). ROS-scavenging-associated transcriptional and biochemical shifts during nectarine fruit development and ripening. Plant Physiol. Biochem. 171, 38–48. doi: 10.1016/j.plaphy.2021.12.022 34971954

[B110] Van Asperen K. (1962). A study of housefly esterases by means of a sensitive colorimetric method. J. Insect Physiol. 8, 401–416. doi: 10.1016/0022-1910(62)90074-4

[B111] Varavinit S. Chaokasem N. Shobsngob S. (2002). Immobilization of a thermostable alpha-amylase. Sci. Asia 28, 247–251. doi: 10.2306/scienceasia1513-1874.2002.28.247

[B112] Vats S. Bansal R. Rana N. Kumawat S. Bhatt V. Jadhav P.. (2020). Unexplored nutritive potential of tomato to combat global malnutrition. Crit. Rev. Food Sci. Nutr. 60, 1–32. doi: 10.1080/10408398.2020.1832954 33086895

[B113] Wang D. Mu Y. Hu X. Ma B. Wang Z. Zhu L.. (2021). Comparative proteomic analysis reveals that the heterosis of two maize hybrids is related to enhancement of stress response and photosynthesis respectively. BMC Plant Biol. 21, 1–15. doi: 10.1186/s12870-020-02806-5 33422018 PMC7796551

[B114] Wargovich M. J. (2000). Anticancer properties of fruits and vegetables. HortScience 35, 573–575. doi: 10.21273/HORTSCI.35.4.573

[B115] Wong F.-C. Xiao J. Wang S. Ee K.-Y. Chai T.-T. (2020). Advances on the antioxidant peptides from edible plant sources. Trends Food Sci. Technol. 99, 44–57. doi: 10.1016/j.tifs.2020.02.012

[B116] Yasui K. Baba A. (2006). Therapeutic potential of superoxide dismutase (SOD) for resolution of inflammation. Inflammation Res. 55, 359–363. doi: 10.1007/s00011-006-5195-y 17122956

[B117] Younus H. (2018). Therapeutic potentials of superoxide dismutase. Int. J. Health Sci. 12, 88. PMC5969776 29896077

[B118] Yu D. Gu X. Zhang S. Dong S. Miao H. Gebretsadik K.. (2021). Molecular basis of heterosis and related breeding strategies reveal its importance in vegetable breeding. Horticul. Res. 8, 120. doi: 10.1038/s41438-021-00552-9 PMC8166827 34059656

[B119] Zhang S. Huang X. Han B. (2021). Understanding the genetic basis of rice heterosis: Advances and prospects. Crop J. 9, 688–692. doi: 10.1016/j.cj.2021.03.011

[B120] Zhang J. Kirkham M. (1994). Drought-stress-induced changes in activities of superoxide dismutase, catalase, and peroxidase in wheat species. Plant Cell Physiol. 35, 785–791. doi: 10.1093/oxfordjournals.pcp.a078658

[B121] Zhong X.-L. Tian Y.-Z. Jia M.-L. Liu Y.-D. Cheng D. Li G. (2020). Characterization and purification *via* nucleic acid aptamers of a novel esterase from the metagenome of paper mill wastewater sediments. Int. J. Biol. Macromol. 153, 441–450. doi: 10.1016/j.ijbiomac.2020.02.319 32119944

[B122] Zhou K. Yu L. (2006). Total phenolic contents and antioxidant properties of commonly consumed vegetables grown in Colorado. LWT Food Sci. Technol. 39, 1155–1162. doi: 10.1016/j.lwt.2005.07.015

