# Supplementary material for: Fruit nutritional composition, antioxidant and biochemical profiling of diverse tomato (Solanum lycopersicum L.) genetic resource
Source: Front Plant Sci. 2022 Oct 13;13:1035163. doi: 10.3389/fpls.2022.1035163 (PMC9608662; doi:10.3389/fpls.2022.1035163)
Supplement: Supplementary file 1 [file DataSheet_1.docx]

**Table S1** **Principal Component Analysis in tomato fruit for different biochemical parameters**

|  | **F1** | **F2** | **F3** | **F4** | **F5** | **F6** | **F7** | **F8** | **F9** | **F10** | **F11** | **F12** | **F13** | **F14** | **F15** | **F16** | **F17** | **F18** | **F19** | **F20** |
| --- | --- | --- | --- | --- | --- | --- | --- | --- | --- | --- | --- | --- | --- | --- | --- | --- | --- | --- | --- | --- |
| **Eigenvalue** | 3.897 | 2.641 | 1.835 | 1.567 | 1.374 | 1.179 | 1.119 | 1.047 | 0.947 | 0.930 | 0.873 | 0.694 | 0.629 | 0.589 | 0.491 | 0.471 | 0.374 | 0.294 | 0.034 | 0.014 |
| **Variability (%)** | 18.557 | 12.578 | 8.737 | 7.463 | 6.544 | 5.612 | 5.329 | 4.988 | 4.508 | 4.431 | 4.155 | 3.305 | 2.994 | 2.803 | 2.339 | 2.245 | 1.779 | 1.402 | 0.163 | 0.069 |
| **Cumulative %** | 18.557 | 31.135 | 39.872 | 47.335 | 53.879 | 59.491 | 64.820 | 69.808 | 74.316 | 78.747 | 82.902 | 86.206 | 89.200 | 92.003 | 94.342 | 96.587 | 98.366 | 99.768 | 99.931 | 100.000 |
| **Factor loading** | | | | | | | | | | | | | | | | | | | | |
|  | **F1** | **F2** | **F3** | **F4** | **F5** | **F6** | **F7** | **F8** | **F9** | **F10** | **F11** | **F12** | **F13** | **F14** | **F15** | **F16** | **F17** | **F18** | **F19** | **F20** |
| Lycopene | 0.450 | -0.007 | 0.127 | -0.147 | -0.010 | -0.116 | -0.011 | -0.018 | -0.128 | -0.061 | 0.059 | 0.008 | 0.161 | 0.085 | 0.220 | 0.041 | -0.348 | 0.073 | 0.714 | 0.049 |
| Chlorophyll a | 0.351 | -0.215 | -0.242 | 0.014 | 0.048 | 0.000 | -0.109 | -0.004 | 0.186 | 0.143 | 0.008 | -0.246 | -0.093 | -0.101 | -0.392 | -0.341 | 0.148 | 0.327 | 0.081 | -0.001 |
| Chlorophyll b | 0.380 | 0.208 | 0.207 | -0.027 | 0.050 | -0.048 | 0.067 | 0.066 | -0.127 | 0.019 | 0.106 | 0.201 | -0.181 | 0.108 | 0.178 | 0.242 | 0.445 | -0.319 | -0.106 | -0.027 |
| Total carotenoids | 0.450 | -0.057 | 0.076 | -0.102 | 0.014 | -0.116 | -0.056 | -0.084 | -0.097 | -0.028 | 0.008 | -0.104 | 0.206 | 0.025 | 0.165 | -0.016 | -0.431 | 0.119 | -0.680 | -0.020 |
| Total chlorophyll | 0.477 | 0.002 | -0.016 | -0.009 | 0.063 | -0.032 | -0.024 | 0.042 | 0.034 | 0.104 | 0.076 | -0.022 | -0.180 | 0.008 | -0.131 | -0.055 | 0.391 | -0.005 | -0.019 | -0.019 |
| TPC^[[1]](#footnote-1)^ | -0.083 | -0.036 | 0.001 | -0.111 | 0.318 | -0.175 | 0.563 | -0.399 | -0.050 | 0.390 | 0.002 | 0.251 | 0.178 | 0.174 | -0.054 | 0.027 | 0.092 | 0.282 | -0.005 | -0.011 |
| Reducing Sugar | 0.113 | 0.504 | -0.265 | 0.184 | -0.051 | 0.077 | 0.127 | -0.046 | 0.133 | -0.152 | -0.078 | 0.064 | 0.017 | -0.041 | 0.031 | 0.040 | -0.012 | 0.184 | -0.037 | 0.715 |
| TSS^[[2]](#footnote-2)^ | 0.085 | 0.512 | -0.143 | 0.242 | 0.006 | 0.000 | 0.015 | 0.076 | 0.233 | -0.085 | -0.077 | 0.244 | 0.014 | -0.070 | 0.092 | -0.081 | -0.083 | 0.262 | 0.026 | -0.650 |
| NRS^[[3]](#footnote-3)^ | -0.106 | -0.071 | 0.415 | 0.040 | 0.149 | -0.257 | -0.294 | 0.356 | 0.214 | 0.165 | 0.055 | 0.454 | 0.022 | -0.007 | 0.111 | -0.336 | 0.011 | 0.195 | -0.041 | 0.245 |
| Alpha amylase | 0.042 | -0.165 | 0.097 | 0.129 | -0.328 | 0.403 | 0.289 | 0.458 | -0.261 | 0.188 | -0.224 | -0.062 | 0.017 | 0.149 | 0.209 | 0.031 | 0.108 | 0.381 | -0.027 | -0.007 |
| AsA^[[4]](#footnote-4)^ | -0.068 | 0.253 | -0.211 | -0.360 | 0.003 | -0.088 | -0.049 | 0.341 | -0.242 | -0.024 | 0.122 | -0.057 | 0.662 | -0.002 | -0.158 | -0.151 | 0.245 | -0.078 | -0.015 | -0.031 |
| MDA^[[5]](#footnote-5)^ | -0.003 | 0.311 | -0.080 | -0.198 | 0.269 | 0.067 | 0.001 | 0.306 | -0.062 | 0.527 | -0.236 | -0.120 | -0.306 | 0.072 | -0.169 | -0.003 | -0.373 | -0.262 | 0.011 | 0.023 |
| TAC^[[6]](#footnote-6)^ | 0.082 | -0.167 | -0.045 | -0.272 | 0.316 | 0.355 | 0.313 | 0.089 | 0.436 | -0.180 | -0.155 | -0.018 | 0.093 | -0.218 | 0.359 | -0.257 | 0.047 | -0.241 | 0.000 | 0.004 |
| Protease | 0.137 | 0.008 | 0.136 | 0.355 | -0.254 | 0.357 | -0.075 | -0.210 | 0.165 | 0.452 | 0.111 | 0.135 | 0.412 | -0.141 | -0.124 | -0.052 | -0.071 | -0.344 | 0.032 | 0.020 |
| Esterase | 0.058 | -0.276 | -0.345 | -0.074 | 0.015 | 0.044 | 0.051 | 0.338 | 0.292 | 0.020 | 0.374 | 0.307 | 0.019 | -0.004 | -0.149 | 0.550 | -0.167 | 0.053 | -0.033 | 0.006 |
| APX^[[7]](#footnote-7)^ | -0.024 | -0.114 | -0.339 | 0.124 | 0.346 | 0.057 | -0.449 | -0.111 | -0.236 | 0.253 | -0.180 | 0.018 | 0.094 | -0.287 | 0.424 | 0.223 | 0.168 | 0.131 | 0.036 | 0.016 |
| TOS^[[8]](#footnote-8)^ | 0.144 | -0.232 | -0.179 | 0.240 | 0.055 | -0.115 | 0.180 | 0.098 | -0.389 | -0.241 | -0.366 | 0.457 | -0.008 | -0.228 | -0.277 | -0.160 | -0.116 | -0.239 | 0.002 | 0.011 |
| SOD^[[9]](#footnote-9)^ | 0.012 | 0.050 | 0.412 | 0.270 | 0.278 | -0.197 | 0.192 | 0.206 | 0.082 | -0.057 | -0.106 | -0.368 | 0.190 | -0.439 | -0.196 | 0.358 | 0.029 | 0.078 | 0.034 | 0.001 |
| CAT^[[10]](#footnote-10)^ | -0.060 | -0.008 | -0.162 | 0.420 | 0.223 | -0.057 | 0.231 | 0.158 | -0.238 | 0.029 | 0.622 | -0.192 | -0.107 | -0.017 | 0.225 | -0.309 | -0.103 | -0.110 | -0.001 | 0.008 |
| POD^[[11]](#footnote-11)^ | -0.022 | 0.153 | 0.241 | -0.305 | 0.101 | 0.473 | -0.047 | -0.129 | -0.311 | -0.090 | 0.319 | 0.200 | -0.178 | -0.438 | -0.189 | -0.023 | -0.075 | 0.244 | -0.018 | -0.001 |
| TFC^[[12]](#footnote-12)^ | 0.030 | -0.019 | 0.118 | 0.231 | 0.510 | 0.394 | -0.203 | 0.000 | -0.037 | -0.264 | -0.041 | 0.000 | 0.162 | 0.565 | -0.228 | 0.052 | -0.014 | 0.035 | 0.003 | -0.004 |

**Table S2 Factor Score**

| **Factor Score** | **F1** | **F2** | **Factor Score** | **F1** | **F2** | **Factor Score** | **F1** | **F2** |
| --- | --- | --- | --- | --- | --- | --- | --- | --- |
| **Ahmar F1^[[13]](#footnote-13)^** | **3.531** | -0.085 | **17253^[[14]](#footnote-14)^** | -0.307 | -0.963 | **NCEBR-6** | -0.502 | **1.198** |
| **Iron-Lady F1** | **4.924** | -2.392 | **21354** | -0.798 | **3.517** | **PRN-28-10** | **1.178** | -1.237 |
| **NBH-149** | **0.128** | -1.811 | **21396** | -0.119 | -0.729 | **Riogrande** | -1.135 | **0.226** |
| **NBH-150** | -0.463 | **1.114** | **AVTO1009** | **1.165** | **0.558** | **Roma** | **1.314** | **0.028** |
| **NBH-151** | -1.435 | **2.381** | **AVTO1010** | -1.731 | -0.722 | **Galia** | -0.304 | **0.598** |
| **NBH-152** | -1.017 | **1.962** | **AVTO1311** | **2.847** | **1.770** | **B-31** | -1.777 | -1.257 |
| **NBH-154** | -1.726 | -3.234 | **AVTO1315** | **3.522** | **3.469** | **Canada-25** | -1.206 | **0.102** |
| **NBH-182** | -1.277 | 0.782 | **B-L-35** | **0.180** | -0.616 | **Flora-Dade** | -0.822 | -0.644 |
| **NBH-188** | -1.564 | -0.417 | **CLN2768** | -0.178 | -0.034 | **CKD-6-15 F6^[[15]](#footnote-15)^** | **0.975** | **0.393** |
| **NBH-190** | -2.049 | -0.791 | **LA4097** | -0.547 | **1.773** | **CKD-8-15 F6** | **0.083** | -1.638 |
| **NBH-196** | -0.941 | **2.371** | **LA4141** | -1.144 | **0.384** | **MIL-10-F4** | -1.182 | -1.544 |
| **NBH-200** | -0.223 | -2.375 | **LBR-17** | **6.640** | **0.111** | **MIL-13-F4** | -1.079 | **1.329** |
| **NBH-204** | **0.017** | **3.232** | **Lukullus** | **1.357** | -1.612 | **T-1359-6-15F6** | **0.257** | -1.206 |
| **NBH-227** | -1.967 | -0.427 | **Lyp-1** | **1.674** | -2.094 | **Factor Score** ^[[16]](#footnote-16)^ | **F1** | **F2** |
| **NBH-228** | -0.695 | **1.800** | **Money maker** | -0.718 | 0.598 | **Category-Advance line** | -0.189 | -0.533 |
| **NBH-229** | -0.761 | **3.188** | **Nadir** | -1.206 | -1.774 | **Category-Hybrid** | -0.283 | **0.114** |
| **NBH-235** | -2.000 | **1.143** | **New cherry** | -1.259 | **2.525** | **Category-Line** | **0.186** | **0.019** |
| **NBH-255** | -1.371 | **1.719** | **NI-Cherry** | -0.860 | -0.888 | **Category-Parent** | **0.325** | -0.098 |
| **NBH-256** | **0.990** | **1.740** | **Pakit** | -1.402 | -2.265 | **Factor Score** | **F1** | **F2** |
| **NBH-257** | -1.124 | 0.226 | **UAF-1** | -0.084 | **1.579** | **Growth type-Determinate** | **0.015** | -0.124 |
| **NBH-258** | **4.826** | -0.082 | **V-48** | **0.714** | -2.072 | **Growth type-Indeterminate** | -0.470 | **0.252** |
| **NBH-259** | -0.461 | -2.418 | **V-83** | -2.236 | -1.350 | **Growth type-Semi determinate** | **3.184** | **2.619** |
| **NBH-260** | -0.508 | -0.616 | **Vendor** | -0.712 | -1.947 |  |  |  |
| **NBH-261** | **0.233** | **1.954** | **West virginia-63** | -0.343 | **1.234** |  |  |  |
| **NBH-263** | -1.784 | -0.987 | **Astra^[[17]](#footnote-17)^** | **3.140** | -1.824 |  |  |  |
| **NBH-265** | -1.650 | 0.403 | **AVTO 1003** | **1.447** | -1.077 |  |  |  |
| **NBH-266** | -1.829 | 0.363 | **AVTO 1005** | -1.172 | -2.083 |  |  |  |
| **NBH-267** | -1.847 | 0.399 | **AVTO 1080** | **3.314** | -1.031 |  |  |  |
| **NBH-268** | -0.340 | -2.101 | **AVTO 1219** | **0.223** | -0.421 |  |  |  |
| **NBH-281** | -0.994 | -1.567 | **B-23** | -1.299 | **0.074** |  |  |  |
| **NBH-282** | -1.682 | -1.035 | **B-24** | -1.889 | -2.188 |  |  |  |
| **NBH-5** | **0.244** | -1.461 | **B-25** | **5.036** | -1.051 |  |  |  |
| **NBH-78** | -1.614 | -0.211 | **H-24** | **7.104** | **0.696** |  |  |  |
| **NBH-95** | **0.646** | -0.480 | **LA4157** | -1.490 | -0.520 |  |  |  |
| **NIAB-Gohar** | **1.735** | -1.228 | **LBR-10** | -0.625 | **1.524** |  |  |  |
| **NIAB-Jauhar** | **5.232** | **1.742** | **LBR-7** | -2.134 | -1.119 |  |  |  |
| **Sahel F1** | -1.044 | **0.679** | **M-82** | -0.269 | **1.729** |  |  |  |
| **Sundar F1** | -1.676 | -0.737 | **Nagina** | **1.215** | -0.282 |  |  |  |
| **Surkhail F1** | -0.142 | **3.378** | **Naqeeb** | -0.317 | **3.250** |  |  |  |
| **T-1359 F1** | **0.364** | -1.547 | **NCEBR-5** | -1.225 | **2.949** |  |  |  |

**Table S3 Correlation matrix (Pearson (n)) for biochemical traits in tomato genotypes.**

| **Variables** | **Lycopene** | **Chlorophyll a** | **Chlorophyll b** | **Total carotenoids** | **Total chlorophyll** | **TPC** | **Reducing Sugar** | **Total soluble**  **sugars** | **N.R.S.** | **Alpha amlyase** | **As.A** | **MDA** | **TAC** | **Protease** | **Esterase** | **APX** | **TOS** | **SOD** |
| --- | --- | --- | --- | --- | --- | --- | --- | --- | --- | --- | --- | --- | --- | --- | --- | --- | --- | --- |
| **Lycopene** | **1** |  |  |  |  |  |  |  |  |  |  |  |  |  |  |  |  |  |
| **Chlorophyll a** | **0.455** | **1** |  |  |  |  |  |  |  |  |  |  |  |  |  |  |  |  |
| **Chlorophyll b** | **0.685** | 0.175 | **1** |  |  |  |  |  |  |  |  |  |  |  |  |  |  |  |
| **Total carotenoids** | **0.944** | **0.559** | **0.574** | **1** |  |  |  |  |  |  |  |  |  |  |  |  |  |  |
| **Total chlorophyll** | **0.747** | **0.753** | **0.780** | **0.739** | **1** |  |  |  |  |  |  |  |  |  |  |  |  |  |
| **TPC** | -0.097 | -0.124 | -0.064 | -0.099 | -0.122 | **1** |  |  |  |  |  |  |  |  |  |  |  |  |
| **Reducing Sugar** | 0.073 | -0.030 | **0.301** | 0.041 | 0.182 | -0.094 | **1** |  |  |  |  |  |  |  |  |  |  |  |
| **Total soluble sugars** | 0.045 | -0.101 | **0.301** | 0.000 | 0.137 | -0.128 | **0.923** | **1** |  |  |  |  |  |  |  |  |  |  |
| **NRS** | -0.089 | **-0.210** | -0.016 | -0.135 | -0.144 | -0.042 | **-0.395** | -0.061 | **1** |  |  |  |  |  |  |  |  |  |
| **Alpha-amlyase** | 0.041 | 0.027 | 0.027 | 0.011 | 0.035 | -0.139 | -0.159 | -0.187 | -0.044 | **1** |  |  |  |  |  |  |  |  |
| **As.A** | -0.034 | -0.159 | -0.028 | -0.084 | -0.120 | -0.023 | **0.232** | 0.186 | -0.075 | -0.102 | **1** |  |  |  |  |  |  |  |
| **MDA** | -0.040 | -0.054 | 0.146 | -0.065 | 0.063 | 0.098 | **0.276** | **0.310** | 0.008 | -0.032 | **0.295** | **1** |  |  |  |  |  |  |
| **TAC** | 0.112 | **0.218** | -0.009 | 0.125 | 0.133 | 0.129 | -0.108 | -0.142 | -0.071 | 0.075 | -0.056 | 0.002 | **1** |  |  |  |  |  |
| **Protease** | 0.134 | 0.170 | 0.152 | 0.176 | **0.210** | -0.067 | 0.099 | 0.132 | -0.008 | **0.221** | **-0.248** | -0.119 | -0.103 | **1** |  |  |  |  |
| **Esterase** | 0.033 | **0.320** | -0.109 | 0.037 | 0.131 | -0.033 | -0.167 | -0.191 | -0.056 | 0.082 | 0.013 | -0.119 | **0.239** | -0.049 | **1** |  |  |  |
| **APX** | -0.121 | 0.166 | -0.185 | -0.026 | -0.018 | -0.006 | -0.067 | -0.069 | -0.072 | -0.135 | 0.003 | 0.088 | -0.007 | -0.013 | 0.128 | **1** |  |  |
| **TOS** | 0.180 | **0.278** | 0.058 | **0.213** | **0.215** | 0.021 | -0.066 | -0.094 | -0.106 | 0.191 | -0.160 | **-0.205** | 0.047 | -0.033 | 0.176 | 0.171 | **1** |  |
| **SOD** | 0.037 | -0.121 | 0.148 | 0.047 | 0.022 | 0.047 | -0.041 | 0.058 | **0.274** | 0.014 | -0.141 | 0.021 | 0.004 | 0.044 | **-0.228** | -0.153 | 0.001 | **1** |

Values in bold are different from 0 with a significance level alpha=0.05


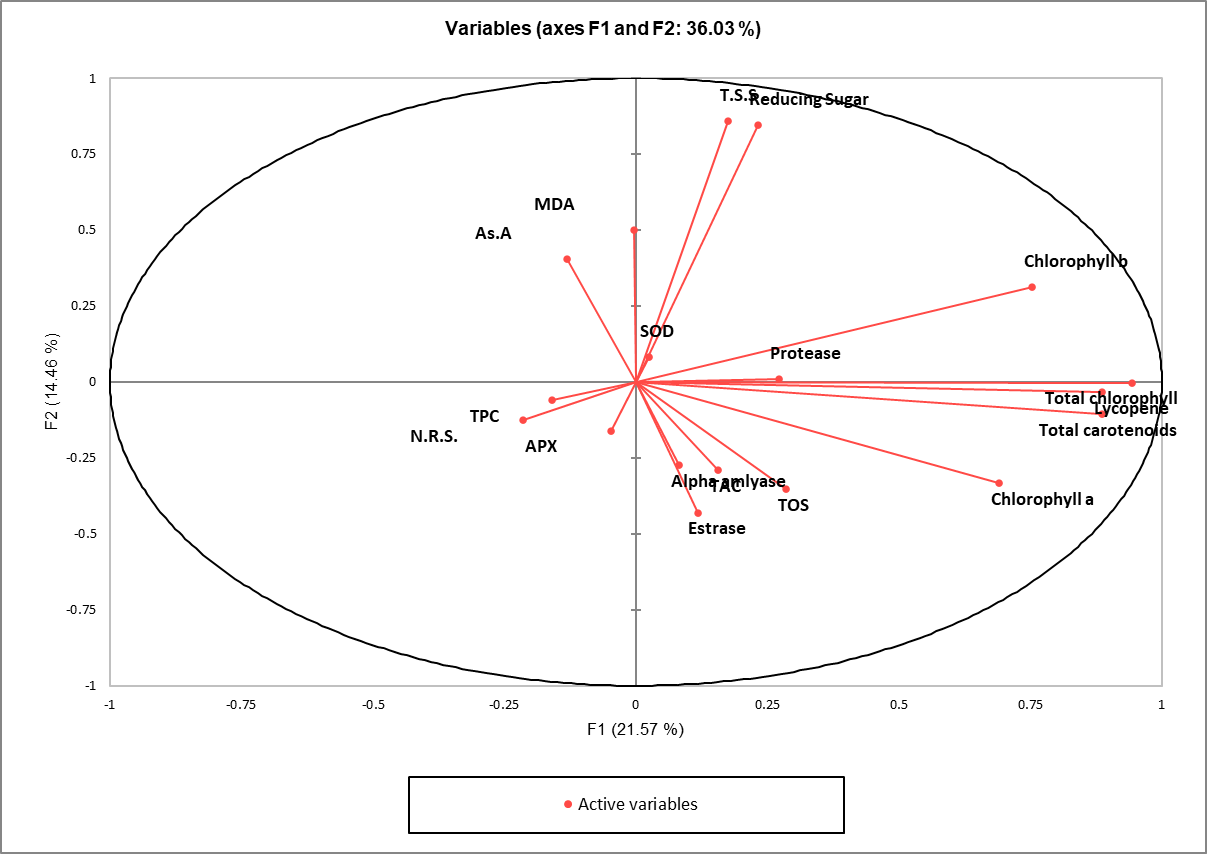


**Figure S1 Correlation circle**

1. Total phenolic compounds, [↑](#footnote-ref-1)
2. Total soluble sugars [↑](#footnote-ref-2)
3. Non reducing sugars [↑](#footnote-ref-3)
4. Ascorbic Acid [↑](#footnote-ref-4)
5. Malondialdehyde [↑](#footnote-ref-5)
6. Total antioxidant capacity [↑](#footnote-ref-6)
7. Ascorbate per oxidase [↑](#footnote-ref-7)
8. Total oxidant status [↑](#footnote-ref-8)
9. Superoxide dismutase [↑](#footnote-ref-9)
10. Catalase [↑](#footnote-ref-10)
11. Peroxidase [↑](#footnote-ref-11)
12. Total flavonoid content [↑](#footnote-ref-12)
13. Hybrids [↑](#footnote-ref-13)
14. Lines [↑](#footnote-ref-14)
15. Advance lines [↑](#footnote-ref-15)
16. **Centroids of the categories** [↑](#footnote-ref-16)
17. Parents [↑](#footnote-ref-17)
